# Incidence of and Neurodevelopmental Outcomes After Late-Onset Meningitis Among Children Born Extremely Preterm

**DOI:** 10.1001/jamanetworkopen.2022.45826

**Published:** 2022-12-08

**Authors:** Jane E. Brumbaugh, Edward F. Bell, Barbara T. Do, Rachel G. Greenberg, Barbara J. Stoll, Sara B. DeMauro, Heidi M. Harmon, Susan R. Hintz, Abhik Das, Karen M. Puopolo

**Affiliations:** 1 Department of Pediatric and Adolescent Medicine, Mayo Clinic, Rochester, Minnesota; 2 Department of Pediatrics, University of Iowa, Iowa City; 3 Social, Statistical and Environmental Sciences Unit, RTI International, Research Triangle Park, North Carolina; 4 Department of Pediatrics, Duke University, Durham, North Carolina; 5 Emory University School of Medicine, Department of Pediatrics, Children’s Healthcare of Atlanta, Atlanta, Georgia; 6 McGovern Medical School of UTHealth, Houston, Texas; 7 Department of Pediatrics, University of Pennsylvania, Philadelphia; 8 Department of Pediatrics, Division of Neonatal and Developmental Medicine, Stanford University School of Medicine and Lucile Packard Children’s Hospital, Palo Alto, California; 9 Social, Statistical and Environmental Sciences Unit, RTI International, Rockville, Maryland

## Abstract

**Importance:**

Late-onset meningitis (LOM) has been associated with adverse neurodevelopmental outcomes in children born extremely preterm.

**Objective:**

To report the incidence of LOM during birth hospitalization and neurodevelopmental outcomes at 18 to 26 months’ corrected age.

**Design, Setting, and Participants:**

This cohort study is a secondary analysis of a multicenter prospective cohort of children born at 22 to 26 weeks’ gestation between 2003 and 2017 with follow-up from 2004 to 2021. The study was conducted at 25 Eunice Kennedy Shriver National Institute of Child Health and Human Development Neonatal Research Network centers.

**Exposures:**

Culture-confirmed LOM.

**Main Outcomes and Measures:**

Incidence and microbiology of LOM (2003-2017); lumbar puncture (LP) performance in late-onset sepsis (LOS) evaluations (2011-2017); composite outcome of death or neurodevelopmental impairment (NDI; 2004-2021).

**Results:**

Among 13 372 infants (median [IQR] gestational age, 25.4 [24.4-26.1] weeks; 6864 [51%] boys), LOM was diagnosed in 167 (1%); LOS without LOM in 4564 (34%); and neither LOS nor LOM in 8641 (65%). The observed incidence of LOM decreased from 2% (95% CI, 1%-3%) in 2003 to 0.4% (95% CI, 0.7%-1.0%) in 2017 (*P* < .001). LP performance in LOS evaluations decreased from 36% (95% CI, 33%-40%) in 2011 to 24% (95% CI, 21%-27%) in 2017 (*P* < .001). Among infants with culture-confirmed LOS, LP performance decreased from 58% (95% CI, 51%-65%) to 45% (95% CI, 38%-51%; *P* = .008). LP performance varied by center among all LOS evaluations (10%-59%, *P* < .001) and among those with culture-confirmed LOS (23%-79%, *P* < .001). LOM occurred in the absence of concurrent LOS in 27 of 167 cases (16%). The most common LOM isolates were coagulase-negative *Staphylococcus* (98 [59%]), *Candida albicans* (38 [23%]), and *Escherichia coli* (27 [16%]). Death or NDI occurred in 22 of 46 children (48%) with LOM due to coagulase-negative *Staphylococcus*, 43 of 67 (64%) due to all other bacterial pathogens, and 26 of 33 (79%) due to fungal pathogens. The adjusted relative risk of death or NDI was increased among children with LOM (aOR, 1.53; 95% CI, 1.04-2.25) and among those with LOS without LOM (aOR, 1.41; 95% CI, 1.29-1.54) compared with children with neither infection.

**Conclusions and Relevance:**

In this cohort study, LP was performed with decreasing frequency, and the observed incidence of LOM also decreased. Both LOM and LOS were associated with increased risk of death or NDI; risk varied by LOM pathogen. The full association of LOM with outcomes of children born extremely preterm may be underestimated by current diagnostic practices.

## Key Points

**Question:**

What is the incidence of and outcomes following late-onset meningitis in children born extremely preterm?

**Findings:**

In this cohort study of 13 372 children born extremely preterm, 1% of children were diagnosed with late-onset meningitis, and 16% of these cases occurred in the absence of a concurrent positive blood culture. Those affected by late-onset meningitis had a high incidence of death or neurodevelopmental impairment.

**Meaning:**

The association of late-onset meningitis with death or neurodevelopmental impairment highlights the importance of lumbar puncture during the evaluation of late-onset infection.

## Teaser

This cohort study reports the incidence of late-onset meningitis during birth hospitalization of neonates born extremely preterm and the neurodevelopmental outcomes at 18 to 26 months’ corrected age.

## Introduction

The newborn period is associated with a higher incidence of meningitis than any other age.^1,2,3^ The immature immune system and permeability of the blood-brain barrier put newborns at increased risk of meningitis.^4^ Whether to perform a lumbar puncture (LP) to obtain cerebrospinal fluid (CSF) as part of a late-onset sepsis (LOS) evaluation in infants born extremely preterm remains an area of debate.^5^ The incidence of culture-confirmed neonatal meningitis is approximately 0.3 per 1000 live births in developed countries.^6^ This may be an underestimation due to the variability in which CSF is obtained in evaluations for LOS. A prospective study found that CSF was 5 times more likely to be positive in LOS than in early-onset sepsis.^7^ With the incidence of LOS decreasing in recent decades,^8^ a question remains whether the incidence of late-onset meningitis (LOM) is declining.

Using data from 1998 to 2001, Stoll et al^9^ found that 1.4% of very low birth weight infants had LOM. The complications of LOM were notable: 23% of the infants died, and affected infants were 14 times more likely to experience seizures than unaffected infants. The rationale for incorporating LP as part of LOS evaluation is that the procedure is safe, effective treatment exists for meningitis, and the implications of missing the diagnosis of meningitis are grave.^10^ Early childhood neurological morbidities associated with meningitis among survivors include cerebral palsy, blindness, hearing loss, seizure disorder, microcephaly, and cognitive impairment.^11,12^

Antibiotic use has evolved since LOM was last systematically studied within the Eunice Kennedy Shriver National Institute of Health and Human Developmental Neonatal Research Network (NRN).^9,13^ The objectives of the current study were to report the detected incidence of LOM, the neurodevelopmental outcomes of infants affected by LOM, and the frequency of LP during LOS evaluations.

## Methods

### Study Period and Definitions

This cohort study was a secondary analysis of prospectively collected data for a cohort of children born at 22 to 26 weeks’ gestation and cared for at 25 NRN centers from 2003 to 2017. Data collection for the databases maintained by the NRN were approved by each site’s institutional review board (IRB); parental consent was obtained if required by the local IRB. Data were reported in accordance with Strengthening the Reporting of Observational Studies in Epidemiology (STROBE) guideline.^14^ Analyses of LOM incidence and outcomes used data collected from 2003 to 2017. Analyses of LP performance used data collected from 2011 to 2017 because data regarding LP performance as part of LOS evaluations were not available before 2011. NRN centers did not share a standard protocol for the use of LP in LOS evaluations.

LOS was defined as isolation of a bacterial or fungal pathogen from blood obtained more than 72 hours after birth and accompanied by treatment for at least 5 days or death before completed treatment. LOM was defined as isolation of a bacterial or fungal pathogen from CSF culture obtained more than 72 hours after birth and accompanied by treatment for at least 7 days or death before completed treatment. Coagulase-negative *Staphylococcus* (CoNS) was only considered a pathogen in the CSF if concurrently identified in the CSF and blood. CSF and blood cultures were considered concurrent if obtained within 7 days of each other. For classification of infectious episodes, if different organisms were cultured within 7 days, then the infection was considered polymicrobial. If a different organism was cultured more than 7 days from the original culture, then the second was considered a new infection. If the same organism was cultured after 10 days of appropriate antibiotic therapy, then it was considered a new infection.

Exclusion criteria were death before 72 hours after birth, early-onset meningitis (≤72 hours after birth), major congenital anomalies, presence of a ventricular shunt or reservoir, viral LOM, polymicrobial LOM without concurrent LOS with 1 of the same pathogens, and missing LOS or LOM data. LOM and LOS data were missing for only 0.1% (17 of 17 858 infants); they were considered missing at random and excluded from analyses. Outcomes included LOS, LP performance as part of LOS evaluation (2011-2017), and the composite outcome of neurodevelopmental impairment (NDI) or death before follow-up. Intracranial hemorrhage (ICH) was classified by Papile criteria^15^ with severe hemorrhage defined as grades 3 and 4.

### Neurodevelopmental Assessment

To reduce loss to follow-up and minimize bias, NRN centers attempted to maintain contact between discharge and follow-up. Follow-up occurred at 18 to 22 months’ corrected age for infants born prior to July 2012 and at 22 to 26 months’ corrected age for those born in July 2012 or later. Bayley Scales of Infant and Toddler Development were used for cognitive, language, and motor skills assessment. The second edition (BSID-II) was used from 2003 to 2007.^16^ The third edition (Bayley-III) was used from 2007 to 2017.^17^ Cerebral palsy was determined by standardized neurological examination with severity assigned using a modification of the Palisano Gross Motor Function Classification System.^18^ NDI included the presence of one or more of the following: moderate to severe cerebral palsy with gross motor function classification level of 2 or greater, BSID-II mental development index of less than 70 or Bayley-III cognitive composite score of less than 85, bilateral blindness (visual acuity <20/200), or bilateral hearing impairment with no functional hearing (with or without amplification). This definition has been used previously^19^ and accounts for the differences in the BSID-II and Bayley-III estimation of cognitive performance.^20,21,22,23^ Death after discharge without NDI assessment was determined from medical records to reduce loss to follow-up.

### Statistical Analysis

Demographic and perinatal characteristics, morbidities, and outcomes were compared with χ^2^ tests for categorical variables and Kruskal-Wallis tests for continuous variables. Logistic regression modeling was performed to determine the association of the following covariates with LOM with adjustment for center: birth year, gestational age, multiple gestation, maternal insurance, antenatal steroid exposure, sex, small-for-gestational-age status, and severe ICH. Logistic regression modeling was performed to determine whether NDI or death differed between infants with LOM, infants with LOS without LOM, and infants unaffected by LOS or LOM, with adjustment for center and the previously identified covariates. The Cochran-Armitage test was used to evaluate for a trend in the incidence of LOM (2003-2017) and for a trend in the performance of LP (2011-2017). Two-sided *P* < .05 was considered statistically significant. Analyses were performed using SAS version 9.4 (SAS Institute).

## Results

### Clinical Characteristics of Cohort

**Figure 1.**
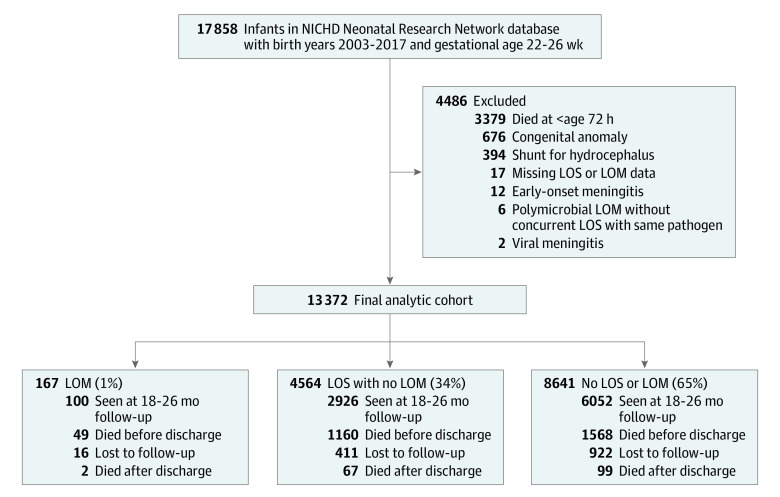
Study Flow Diagram The cohort was broken into 3 groups, those affected by late-onset meningitis (LOM), those affected by late-onset sepsis (LOS) without LOM, and those affected by neither LOS nor LOM. NICHD indicates National Institute of Child Health and Human Development.

Among 13 372 infants (6864 [51%] boys) with a median (IQR) gestational age of 25.4 (24.4-26.1) weeks, 167 (1%) had LOM, 4564 (34%) had LOS without LOM, and 8641 (65%) had neither LOS nor LOM (Figure 1). Across these 3 groups, mothers of infants with LOM were younger and less likely to deliver by cesarean section, have private insurance, have a college degree, or have a hypertensive disorder (Table 1). Infant outborn status, sex, gestational age, and birth weight differed across groups. Infants with either LOM or LOS had a lower gestational age and birth weight than infants with neither infection. Multiple morbidities, including severe ICH and periventricular leukomalacia, were more common among infants affected by LOM (eTable 1 in Supplement 1). On multivariate analysis, later birth year within the study period and severe ICH remained significantly associated with LOM (eTable 2 in Supplement 1).

**Table 1.** Maternal and Neonatal Characteristics

Characteristics	Infants with LOM, No./total No. (%) (n = 167)	Infants with LOS and no LOM, No./total No. (%) (n = 4564)	Infants with no LOS or LOM, No./total No. (%) (n = 8641)	*P* value^a^
**Maternal**				
Maternal age, median (IQR), y^b^	26.0 (22.0-31.0)	27.0 (22.0-32.0)	27.0 (23.0-32.0)	.002
Maternal education				
<High school	34/124 (27)	851/3437 (25)	1468/6483 (23)	.002
High school degree	37/124 (30)	1087/3437 (32)	1914/6483 (30)	
Partial college, trade, technical	34/124 (27)	790/3437 (23)	1608/6483 (25)	
≥College degree	19/124 (15)	709/3437 (21)	1493/6483 (23)	
Private medical insurance	51/154 (33)	1646/4214 (39)	3384/7812 (43)	<.001
Race				
Black	63/166 (38)	1908/4485 (43)	3524/8465 (42)	.17
White	95/166 (57)	2352/4485 (52)	4439/8465 (52)	
Other^c^	8/166 (5)	225/4485 (5)	502/8465 (6)	
Hispanic	28/161 (17)	816/4441 (18)	1325/8397 (16)	<.001
Maternal diabetes	8/166 (5)	211/4549 (5)	395/8613 (5)	.98
Maternal hypertension	22/166 (13)	950/4553 (21)	1891/8609 (22)	.01
Histological chorioamnionitis^d^	61/115 (53)	1511/3112 (49)	3322/6788 (49)	.63
Antenatal steroids	145/165 (88)	3856/4545 (85)	7461/8608 (87)	.01
Antibiotics within 72 h of delivery^d^	89/94 (95)	2215/2416 (92)	4980/5440 (92)	.55
Multiple gestation	40/167 (24)	1106/4564 (24)	2055/8641 (24)	.85
Cesarean section	90/167 (54)	2723/4561 (60)	5482/8629 (64)	<.001
**Neonatal**				
Outborn	12/167 (7)	399/4564 (9)	588/8641 (7)	<.001
Sex				
Female	86/167 (51)	2063/4561 (45)	4365/8640 (50)	<.001
Male	81/167 (49)	2498/4561 (55)	4285/8640 (50)	
Gestational age, median (IQR), wk^e^	25.0 (24.1-25.9)	25.0 (24.1-25.9)	25.6 (24.6-26.3)	<.001
Birth weight, median (IQR), g^e^	699 (605-838)	700 (600-810)	750 (636-870)	<.001
Small for gestational age	9/167 (5)	330/4561 (7)	566/8640 (7)	.26

Abbreviations: LOM, late-onset meningitis; LOS, late-onset sepsis.

^a^ *P* values were calculated from χ^2^ tests for categorical variables and Kruskal-Wallis tests for continuous variables.

^b^ Data on maternal age were available for 167 participants in the LOM cohort, 4562 in the LOS without LOM cohort, and 8636 in the no LOS or LOM cohort.

^c^ Races included in other were American Indian or Alaskan Native (82 participants); Asian, Native Hawaiian, or other Pacific Islander (483 participants); more than 1 race (107 participants); and unknown race (63 participants).

^d^ Available after 2006.

^e^ Data on gestational age and birth weight were available for 167 participants in the LOM cohort, 4564 participants in the LOS without LOM cohort, and 8641 participants in the no LOS or LOM cohort.

### Epidemiology and Microbiology of LOM

**Figure 2.**
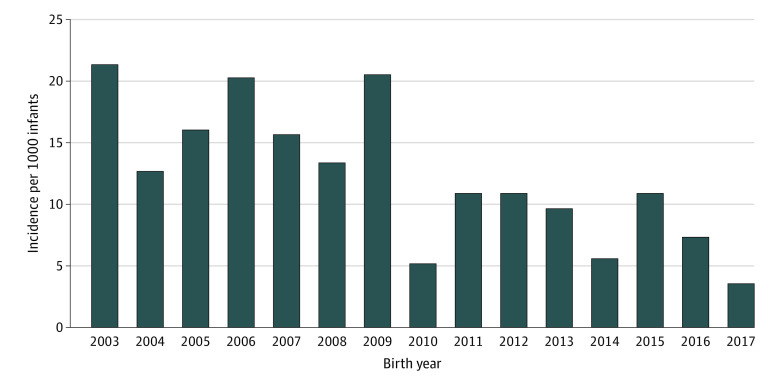
Incidence of Late-Onset Meningitis by Birth Year The incidence of late-onset meningitis decreased across the study period, 2003 to 2017.

**Figure 3.**
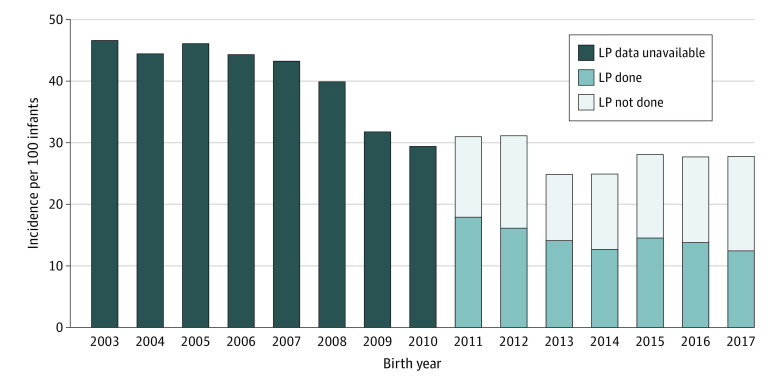
Incidence of Late-Onset Sepsis From 2003 to 2017 and Lumbar Puncture (LP) Among Those With Late-Onset Sepsis From 2011 to 2017 While the incidence of late-onset sepsis decreased from 2003 to 2017, it was relatively stable from 2011 to 2017. However, performance of LP among infants affected by late-onset sepsis decreased from 2011 to 2017.

The incidence of LOM decreased from 2003 (2%; 95% CI, 1%-3%) to 2017 (0.4%; 95% CI, 0.07%-1.0%; *P* < .001) (Figure 2), as did the incidence of LOS (47%; 95% CI, 43%-50% to 28%; 95% CI, 25%-31%; *P* < .001) (Figure 3). Among those with LOM, 27 cases (16%) occurred in the absence of a concurrent positive blood culture, of which 18 (11%) occurred in the absence of any positive blood culture during the hospitalization. Among the 167 infants with LOM, the median (IQR) age for LOM diagnosis was 16 (10-31) days. The incidence of LOM varied across centers (range 0%-7%; *P* < .001) (eFigure 1 in Supplement 1). The most common pathogens in the CSF were CoNS (98 [59%]), *Candida albicans* (38 [23%]), *Escherichia coli* (27 [16%]), *Klebsiella* spp (15 [9%]), group B *Streptococcus* (GBS; 15 [9%]), and *Enterococcus* spp (15 [9%]) (eTable 3 in Supplement 1). For cases of non-CoNS LOM with concurrent LOS, 73 of 96 infants (76%) demonstrated concordance of pathogens in the CSF and blood. Among those with LOM without a concurrent positive blood culture, the most common CSF pathogens were *Candida albicans* and *Staphylococcus aureus*.

### Use of LP in LOS Evaluation

The rate at which LP was performed as part of LOS evaluation decreased from 36% (95% CI, 33%-40%) in 2011 to 24% (95% CI, 21%-27%) in 2017 (*P* < .001). During this period, 1771 of 5865 infants (30%) had LP performed as part of LOS evaluation, and 47 of 1771 (3%) had LOM. Performance of LP as a component of LOS evaluation varied across centers (range 10%-59%; *P* < .001) (eFigure 2 in Supplement 1). Among infants with LOS, LP performance also varied by center (range 23%-79%; *P* < .001) (eFigure 3 in Supplement 1). The incidence of LOS was relatively stable from 2011 (31%; 95% CI, 28%-34%) to 2017 (28%; 95% CI, 25%-31%; *P* = .09) while the performance of LP among infants with LOS decreased from 58% (95% CI, 51%-65%) to 45% (95% CI, 38%-51%; *P* = .008) (Figure 3). LP performance varied by LOS pathogen, ranging from 42% (26 of 62) for *Pseudomonas* LOS to 83% (50 of 60) for GBS LOS (eTable 4 in Supplement 1).

### Neurodevelopmental Outcomes and Death

NDI assessment at 18 to 26 months’ corrected age or death after discharge (prior to planned assessment) was known for 12 023 of 13 372 children (90%); the remainder were lost to follow-up. The median (IQR) age of death among the 49 infants with LOM who died was 41 (19-124) days. Among survivors, NDI was present in 42% (95% CI, 32%-52%) of children with LOM and in 43% (95% CI, 41%-45%) of children with LOS without LOM compared with only 33% (95% CI, 32%-34%) of children with neither infection (*P* < .001) (Table 2). The incidences of cerebral palsy (24%; 95% CI, 3%-14%), abnormal visual acuity (24%; 95% CI, 15%-32%), and bilateral hearing impairment (8%; 95% CI, 3%-14%) were highest among infants with a history of LOM (Table 2). The adjusted risk of the composite outcome of death or NDI was highest among children with LOM, although the adjusted risk was higher among both infants with LOM and those with LOS compared with infants with neither (eTable 5 in Supplement 1). Risk varied by LOM-infecting pathogen: the composite outcome of death or NDI was present in 48% (95% CI, 33%-63%) of children with CoNS LOM, 64% (95% CI, 53%-76%) of children with non-CoNS LOM, and 79% (95% CI, 65%-93%) of children with fungal LOM (*P* = .02) (eTable 6 in Supplement 1).

**Table 2.** Neurological, Sensory, and Developmental Outcomes at 18 to 26 Months’ Corrected Age

Outcomes	Infants with LOM, No./total No. (%) (n = 151)	Infants with LOS and no LOM, No./total No. (%) (n = 4153)	Infants with no LOS or LOM, No./total No. (%) (n = 7719)	*P* value^a^
NDI or death	91/146 (62)	2436/4043 (60)	3584/7499 (48)	<.001
Death^b^	51/151 (34)	1227/4153 (30)	1667/7719 (22)	<.001
NDI	40/95 (42)	1209/2816 (43)	1917/5832 (33)	<.001
BSID-II mental development index, median (IQR)^c^	73.0 (49.0-79.0)	76.0 (59.0-87.0)	80.0 (64.0-92.0)	<.001
Bayley-III composite scores, median (IQR)^d^				
Motor	85.0 (74.5-95.5)	85.0 (73.0-97.0)	88.0 (79.0-97.0)	<.001
Cognitive	90.0 (75.0-95.0)	85.0 (75.0-95.0)	90.0 (80.0-100.0)	<.001
Language	83.0 (68.0-94.0)	79.0 (68.0-91.0)	86.0 (74.0-94.0)	<.001
Neurological examination				
Abnormal examination	43/97 (44)	1198/2893 (41)	2147/5980 (36)	<.001
Any cerebral palsy	23/97 (24)	487/2892 (17)	760/5982 (13)	<.001
Moderate to severe cerebral palsy	14/97 (14)	245/2891 (8)	308/5982 (5)	<.001
Gross motor function level ≥2	13/97 (13)	314/2887 (11)	395/5981 (7)	<.001
Bilateral hearing impairment	8/96 (8)	148/2880 (5)	159/5940 (3)	<.001
Eye examination				
Normal visual acuity	74/97 (76)	2352/2893 (81)	5334/5982 (89)	<.001
Wears or prescribed corrective lenses	15/97 (15)	340/2893 (12)	461/5982 (8)	
Other vision abnormality	4/97 (4)	106/2893 (4)	125/5982 (2)	
Blind with some or no useful vision	4/97 (4)	95/2893 (3)	62/5982 (1)	

Abbreviations: Bayley-III, Bayley Scales of Infant and Toddler Development, third edition; BSID-II, Bayley Scales of Infant and Toddler Development, second edition; LOM, late-onset meningitis; LOS, late-onset sepsis; NDI, neurodevelopmental impairment.

^a^ *P* values were calculated from χ^2^ tests for categorical variables and Kruskal-Wallis tests for continuous variables.

^b^ For infants with LOS (either with or without LOM), 553 of 4713 (12%) died within 7 days of LOS diagnosis.

^c^ BSID-II scores were available for 23 children in the LOM cohort, 754 in the LOS without LOM cohort, and 921 in the no LOS or LOM cohort.

^d^ Bayley-III motor scores were available for 48 children in the LOM cohort, 1553 in the LOS without LOM cohort, and 4130 in the no LOS or LOM cohort; cognitive scores were available for 72 children in the LOM cohort, 2041 in the LOS without LOM cohort, and 4920 in the no LOS or LOM cohort; and language scores were available for 73 children in the LOM cohort, 1996 in the LOS without LOM cohort, and 4816 in the no LOS or LOM cohort.

## Discussion

In a large, multicenter cohort of extremely preterm infants born over a 15-year period, 1% of infants were diagnosed with culture-confirmed LOM, while one-third experienced late-onset bloodstream infection. Our study has several key findings. First, LOM was associated with a risk of death or NDI beyond the substantial risk associated with extremely preterm birth alone, with the highest risk associated with fungal meningitis. Second, LOM was diagnosed in the absence of concurrent bacteremia in 16% of cases, underscoring that LOM likely is missed in some cases when LP is not included in LOS evaluation. Moreover, clinicians only variably used LP during evaluations for late-onset infection, even when bacteremia was identified, and use declined during years when the incidence of bacteremia was unchanged. These observations suggest that LOM may be an underrecognized contributor to NDI among extremely preterm infants.

Our study adds to prior studies that have observed an association between meningitis and both death and neurological morbidities. Preterm infants are more vulnerable to the effects of meningitis.^9,24,25,26^ Prospective neonatal meningitis surveillance in France from 2001 to 2007 found that the mortality rate of bacterial meningitis varied by gestational age, with a higher rate among preterm compared with term infants (26% vs 10%; *P* < .01).^24^ In an earlier NRN cohort of infants born from 1998 to 2001, infants with LOM had an 8- to 12-fold increased likelihood of death compared with uninfected infants.^9^ We did not observe a significantly increased likelihood of death among infants with LOM compared with uninfected infants in adjusted analyses; however, our study was limited to extremely low gestation infants, meaning the comparison group was younger, smaller, and had more noninfectious morbidities compared with the earlier NRN study. A more recent Canadian cohort of primarily term infants with meningitis identified adverse outcomes in 74%, including death (7%), infarction on neuroimaging, hydrocephalus, and motor deficits; complications were more common among preterm infants.^26^

Despite evidence that meningitis is associated with adverse outcomes, we observed substantial variation in LP performance as part of LOS evaluations. There are several potential explanations for the reluctance of neonatal clinicians to perform LP. It is possible that, as the incidence of overall late-onset infection declined among NRN centers, concern for LOM also declined. The performance of LP can be associated with hypoxemic episodes, and any infant sick enough to warrant evaluation for late-onset infection may be considered too unstable to tolerate the positioning for LP.^27,28,29^ This may be especially true for infants supported with high-frequency oscillatory ventilation, the technicalities of which make changing infant position problematic. Frequently, CSF obtained from LP is contaminated with red blood cells, and cell counts can be challenging to interpret.^30,31^ Fearing that the opportunity to identify LOM has passed after initiation of antibiotic therapy, clinicians may opt to empirically administer antibiotic courses appropriate to LOM when LOS is identified rather than perform LP. Finally, clinicians may decide that the risk of meningitis with bacteremia due to low-virulence species, such as CoNS, is low (only 32% of infants with CoNS LOS had LP done within 7 days of LOS evaluation). Conversely, clinicians may perceive that the risk from highly virulent species, such as *Pseudomonas*, is high (only 29% of infants with *Pseudomonas* LOS had LP done within 7 days of LOS evaluation) and treat empirically for meningitis. In both cases, the perceived risks of LP may exceed the perceived value of diagnostic certainty.

The findings of this study suggest that clinicians should consider LP when late-onset infection is suspected in the extremely preterm population. In an earlier NRN cohort, one-third of infants with LOM had sterile blood cultures.^9^ In another multicenter cohort, meningitis was diagnosed in 2% of preterm infants who underwent LP, and 30% of those cases had sterile blood cultures.^25^ We found that 27 of 167 cases of LOM (16%) occurred in the absence of concurrent bacteremia. These cases all involved recognized pathogens, as we decided a priori that cases of CoNS in CSF were only considered LOM if a concurrent blood culture grew CoNS. Furthermore, we excluded cases of LOM identified among infants with ventricular reservoirs or shunts for hydrocephalus. In this retrospective analysis, we could not exclude the possibility that concurrent bacteremia was present but not diagnosed due to substandard blood culture technique. Among newborns, meningitis generally is believed to be a metastatic complication of bacteremia. However, there is evidence that this may be less often the case for late-onset infection based on US surveillance studies of GBS. Only 11 of 1277 cases of early-onset GBS infection (1%) were diagnosed by isolation from CSF alone.^32^ In contrast, 84 of 1387 late-onset GBS infections (6%) involved isolation from CSF with sterile blood cultures. It is possible that primary central nervous system infection may be more common beyond the immediate newborn period.

Failure to identify LOM may have contributed to some observations in our cohort. Although we found higher adjusted rates of the composite outcome of death or NDI among survivors associated with either LOM or LOS compared with infants without infection, the adjusted rates of death or NDI as individual outcomes were not different for those with LOM compared with infants without infection given the smaller number of LOM cases. In contrast, the individual outcomes of death and NDI for those affected by LOS without LOM were significantly higher than for infants without infection. Inconsistent use of LP among infants undergoing infectious evaluations may have resulted in misclassification of infants with LOM as either uninfected or bacteremic without meningitis. In this cohort, 43% of infants with LOS and without LOM and 33% of infants without either infection survived and had NDI. We speculate that LOM misclassification could result in suboptimal antimicrobial treatment and failure to identify infants who require more intensive follow-up after neonatal intensive care unit discharge.

A final observation in our study that must be acknowledged is the high proportion of LOM cases due to CoNS. Cohort studies of primarily term infants identify GBS and *E coli* as predominant LOM pathogens.^24,26^ Prior studies have excluded CSF CoNS isolation as presumed contaminants.^26^ Because our study focused on children born at 22 to 26 weeks’ gestation, for whom CoNS bacteremia is recognized as a clinically relevant infection, we included CoNS if isolated from concurrent blood and CSF cultures. However, we noted less severe unadjusted outcomes for children with CoNS LOM compared with children with LOM due to other bacterial or fungal pathogens (eTable 5 in Supplement 1). Prospective studies that carefully consider the context of CoNS isolation may be needed to determine whether CoNS is a clinically important cause of central nervous system infection among extremely preterm infants.

### Limitations and Strengths

This study has limitations, included the restricted time frame for which LP use was available and our reliance on a culture-based definition of LOM. Although culture confirmation is a more rigorous definition, CSF cell counts are commonly used by clinicians for the diagnosis of LOM in the absence of positive cultures, and our approach may have misclassified some infants. Furthermore, we could not exclude the possibility that in some cases the LP was done for noninfectious reasons, such as management of posthemorrhagic hydrocephalus or metabolic investigation. These limitations were balanced by the strengths of this study, including the large number of infants with low gestational ages, a sufficient time span to detect a change in the incidence of LOM, and the high rate of follow-up assessment at 18 to 26 months’ corrected age.

## Conclusions

Meningitis remains a relatively rare but serious infection associated with increased risk of neurological morbidities or death for children born extremely preterm. In this cohort study, we observed time and center-based variation in use of LP that likely contributes to inconsistent LOM diagnosis. To improve LP practice, ultrasonographic guidance may prove a useful tool to facilitate obtaining CSF in a timely manner during sepsis evaluations. Meningitis panels using polymerase chain reactions on CSF specimens may complement CSF cultures and improve diagnostic accuracy when cultures are obtained after initiation of antimicrobial therapy. Prospective studies are needed to determine whether consistent, accurate, and timely LOM diagnosis can improve the outcomes of the most vulnerable preterm population.

## Notes

Supplement 1. **eTable 1.** Newborn Hospitalization Morbidities and Outcomes **eTable 2.** Logistic Regression Model for Risk of Late-Onset Meningitis **eTable 3. **Distribution of Pathogens Identified in Cerebrospinal Fluid **eTable 4. **Rate of Lumbar Puncture Performance by Late-Onset Sepsis Pathogen, 2011-2017 **eTable 5. **Logistic Regression Models for Risk of Neurodevelopmental Impairment or Death **eTable 6. **Outcomes at 18 to 26 Months’ Corrected Age by Late-Onset Meningitis Pathogen Classification **eFigure 1. **Incidence of Detected Late-Onset Meningitis by Center (2003-2017) **eFigure 2. **Incidence of Lumbar Puncture (LP) in Late-Onset Sepsis Evaluations by Center, and Percentage of Infants Diagnosed with Late-Onset Meningitis (2011-2017) **eFigure 3. **Incidence of Lumbar Puncture Among Infants with Culture-confirmed Late-Onset Sepsis by Center (2011-2017)

Supplement 2.
Nonauthor Collaborators

Supplement 3.
Data Sharing Statement
