# Supplementary material for: Incidence of and Neurodevelopmental Outcomes After Late-Onset Meningitis Among Children Born Extremely Preterm
Source: JAMA Netw Open. 2022 Dec 8;5(12):e2245826. doi: 10.1001/jamanetworkopen.2022.45826 (PMC9856224; doi:10.1001/jamanetworkopen.2022.45826)
Supplement: Supplement 1. — eTable 1. Newborn Hospitalization Morbidities and Outcomes eTable 2. Logistic Regression Model for Risk of Late-Onset Meningitis eTable 3. Distribution of Pathogens Identified in Cerebrospinal Fluid eTable 4. Rate of Lumbar Puncture Performance by Late-Onset Sepsis Pathogen, 2011-2017 eTable 5. Logistic Regression Models for Risk of Neurodevelopmental Impairment or Death eTable 6. Outcomes at 18 to 26 Months’ Corrected Age by Late-Onset Meningitis Pathogen Classification eFigure 1. Incidence of Detected Late-Onset Meningitis by Center (2003-2017) eFigure 2. Incidence of Lumbar Puncture (LP) in Late-Onset Sepsis Evaluations by Center, and Percentage of Infants Diagnosed with Late-Onset Meningitis (2011-2017) eFigure 3. Incidence of Lumbar Puncture Among Infants with Culture-confirmed Late-Onset Sepsis by Center (2011-2017) [file jamanetwopen-e2245826-s001.pdf]

Supplemental Online Content

Brumbaugh JE, Bell EF, Do BT, et al; Eunice Kennedy Shriver National Institute of Child Health and Human Development Neonatal Research Network. Incidence of and neurodevelopmental outcomes after late-onset meningitis among children born extremely preterm. *JAMA Netw Open.* 2022;5(12):e2245826. doi:10.1001/jamanetworkopen.2022.45826

eTable 1. Newborn Hospitalization Morbidities and Outcomes

eTable 2. Logistic Regression Model for Risk of Late-Onset Meningitis

eTable 3. Distribution of Pathogens Identified in Cerebrospinal Fluid

eTable 4. Rate of Lumbar Puncture Performance by Late-Onset Sepsis Pathogen, 2011-2017

eTable 5. Logistic Regression Models for Risk of Neurodevelopmental Impairment or Death

eTable 6. Outcomes at 18 to 26 Months' Corrected Age by Late-Onset Meningitis Pathogen Classification

eFigure 1. Incidence of Detected Late-Onset Meningitis by Center (2003-2017)

eFigure 2. Incidence of Lumbar Puncture (LP) in Late-Onset Sepsis Evaluations by Center, and Percentage of Infants Diagnosed with Late-Onset Meningitis (2011-2017)

eFigure 3. Incidence of Lumbar Puncture Among Infants with Culture-confirmed Late-Onset Sepsis by Center (2011-2017)

This supplemental material has been provided by the authors to give readers additional information about their work.

© 2022 Brumbaugh JE et al. *JAMA Network Open.*

eTable 1. Newborn Hospitalization Morbidities and Outcomes

Outcomes	n	Infants with LOM n=167	n	Infants with LOS and No LOM n=4564	n	Infants with No LOS or LOM n=8641	P-value[a]
Early-onset sepsis[b], n (%)	167	3 (2)	4563	84 (2)	8640	219 (3)	0.037
Late-onset blood culture negative clinical infection[c], n (%)	167	91 (54)	4563	2385 (52)	8634	4136 (48)	<0.001
Severe intracranial hemorrhage[d], n (%)	166	51 (31)	4520	911 (20)	8536	1517 (18)	<0.001
Periventricular leukomalacia	166	18 (11)	4533	278 (6)	8548	412 (5)	<0.001
Bronchopulmonary dysplasia[e], n (%)	133	88 (66)	3599	2477 (69)	7172	3917 (55)	<0.001
Necrotizing enterocolitis, n (%)	167	29 (17)	4560	829 (18)	8640	803 (9)	<0.001
Failed hearing screen, n (%)	165	20 (12)	4520	491 (11)	8514	843 (10)	0.166
Retinopathy of prematurity (any stage), n (%)	142	106 (75)	3672	2981 (81)	7114	4951 (70)	<0.001
Intervention for retinopathy of prematurity, n (%)	74	21 (28)	2094	550 (26)	4058	687 (17)	<0.001
Surgery, any, n (%)	167	45 (27)	4564	1390 (30)	8640	1468 (17)	<0.001

[a] P-values were calculated from chi-square tests for categorical variables and Kruskal-Wallis tests for continuous variables.
[b] Early-onset sepsis defined as culture-confirmed bloodstream infection occurring within the first 72 hours and accompanied by ≥5 days of antibiotics.
[c] Episode of late-onset infection occurring after 72 hours treated with antibiotics for ≥5 days during the newborn hospitalization.
[d] Severe intracranial hemorrhage was defined as Papile grade III or IV.[15]
[e] Bronchopulmonary dysplasia was defined as respiratory support at 36 weeks' postmenstrual age.

© 2022 Brumbaugh JE et al. *JAMA Network Open.*

eTable 2. Logistic Regression Model for Risk of Late-Onset Meningitis

	Unadjusted results			Adjusted results[a]	
**Characteristics**	**OR (95% CI)**	**P-value**		**OR (95% CI)**	**P-value**
Gestational age (weeks)	0.80 (0.70-0.92)	0.002		0.86 (0.74-1.01)	0.059
Birth year	0.92 (0.89-0.96)	<0.001		0.93 (0.89-0.97)	0.002
Multiple gestation	1.00 (0.70-1.43)	0.997		1.18 (0.81-1.72)	0.387
Private medical insurance	0.69 (0.49-0.97)	0.030		0.76 (0.53-1.08)	0.127
Antenatal steroids	1.18 (0.74-1.88)	0.499		1.51 (0.90-2.53)	0.122
Male	0.89 (0.66-1.21)	0.460		0.87 (0.63-1.20)	0.392
Small for gestational age	0.78 (0.40-1.54)	0.476		0.81 (0.39-1.67)	0.561
Severe ICH[b] (grade III or IV)	1.94 (1.39-2.71)	<0.001		1.81 (1.26-2.61)	0.001

[a] Odds ratio and p-values are from logistic regression model which included all covariates listed in table and center.
[b] ICH, intracranial hemorrhage.

© 2022 Brumbaugh JE et al. *JAMA Network Open.*

eTable 3. Distribution of Pathogens Identified in Cerebrospinal Fluid

	Infants with late-onset meningitis[a] (n=167)	
**Pathogen**	n	%
**Bacterial**		
Coagulase-negative *Staphylococcus*	98	58.7
Escherichia coli	27	16.2
Klebsiella spp.	15	9.0
Group B *Streptococcus*	15	9.0
Enterococcus spp.	15	9.0
Staphylococcus aureus	14	8.4
Streptococcus viridans	4	2.4
Serratia spp.	3	1.8
Other	3	1.8
**Fungal**		
Candida albicans	38	22.8
Candida parapsilosis	6	3.6
Other	1	0.6

[a] Polymicrobial late-onset meningitis was included in analyses if concurrent late-onset sepsis with the same pathogen was identified.

© 2022 Brumbaugh JE et al. *JAMA Network Open.*

eTable 4. Rate of Lumbar Puncture Performance by Late-Onset Sepsis Pathogen, 2011-2017

LOS Pathogen	LOS Cases[a] n	Lumbar Puncture Performed Ever n (%)	Lumbar Puncture within 7 Days of LOS n (%)
**Gram positive bacteria**			
Coagulase negative *Staphylococcus*	716	332 (46)	227 (32)
Staphylococcus aureus	261	135 (52)	107 (41)
Enterococcus	61	34 (56)	24 (39)
Group B *Streptococcus*	60	50 (83)	43 (72)
Other Gram positive bacteria	28	12 (43)	4 (14)
**Gram negative bacteria**			
Escherichia coli	137	68 (50)	55 (40)
Klebsiella	90	56 (62)	43 (48)
Pseudomonas	62	26 (42)	18 (29)
Enterobacter	37	29 (78)	23 (62)
Other Gram negative bacteria	40	26 (65)	18 (45)
**Fungus**			
Candida	61	40 (66)	23 (38)
Other fungus	2	1 (50)	1 (50)
**Other pathogen**			
Other bacteria Gram stain unknown	12	7 (58)	6 (50)
Other	17	9 (53)	7 (41)

[a] Excludes polymicrobial LOS.

© 2022 Brumbaugh JE et al. *JAMA Network Open.*

eTable 5. Logistic Regression Models for Risk of Neurodevelopmental Impairment or Death

	Unadjusted results			Adjusted results[a]	
**Characteristics**	**OR (95% CI)**	**P-value**		**OR (95% CI)**	**P-value**
**Model for risk of NDI[b] or death (composite)**					
Gestational age (weeks)	0.61 (0.59-0.63)	<0.001		0.62 (0.60-0.65)	<0.001
Birth year	0.99 (0.98-1.00)	0.103		1.00 (0.99-1.01)	0.580
Multiple gestation	1.05 (0.97-1.15)	0.232		1.14 (1.03-1.26)	0.010
Private medical insurance	0.62 (0.58-0.67)	<0.001		0.64 (0.58-0.70)	<0.001
Antenatal steroids	0.59 (0.53-0.66)	<0.001		0.81 (0.71-0.92)	0.002
Male	1.45 (1.35-1.56)	<0.001		1.45 (1.33-1.58)	<0.001
Small for gestational age	2.75 (2.34-3.22)	<0.001		3.84 (3.22-4.58)	<0.001
Severe ICH[c] (grade III or IV)	3.57 (3.22-3.96)	<0.001		3.15 (2.80-3.54)	<0.001
Infection status					
LOM[d]	1.81 (1.29-2.53)	0.001		1.53 (1.04-2.25)	0.032
LOS[e] and no LOM[d]	1.66 (1.53-1.79)	<0.001		1.41 (1.29-1.54)	<0.001
No LOS[e] or LOM[d]	REF			REF	
**Model for risk of NDI[b]**					
Gestational age (weeks)	0.72 (0.69-0.75)	<0.001		0.72 (0.68-0.76)	<0.001
Birth year	1.03 (1.01-1.04)	<0.001		1.02 (1.00-1.03)	0.017
Multiple gestation	1.03 (0.93-1.14)	0.525		1.14 (1.02-1.28)	0.025
Private medical insurance	0.62 (0.56-0.68)	<0.001		0.64 (0.57-0.71)	<0.001
Antenatal steroids	0.69 (0.61-0.79)	<0.001		0.86 (0.74-1.00)	0.052
Male	1.45 (1.33-1.59)	<0.001		1.46 (1.33-1.62)	<0.001
Small for gestational age	2.05 (1.70-2.48)	<0.001		2.56 (2.08-3.15)	<0.001
Severe ICH[c] (grade III or IV)	2.23 (1.97-2.52)	<0.001		2.09 (1.82-2.40)	<0.001
Infection status					
LOM[d]	1.49 (0.98-2.24)	0.059		1.26 (0.79-2.01)	0.337
LOS[e] and no LOM[d]	1.54 (1.40-1.69)	<0.001		1.34 (1.20-1.49)	<0.001
No LOS[e] or LOM[d]	REF			REF	
**Model for risk of death**					
Gestational age (weeks)	0.57 (0.55-0.59)	<0.001		0.58 (0.55-0.61)	<0.001
Birth year	0.95 (0.94-0.96)	<0.001		0.96 (0.95-0.97)	<0.001
Multiple gestation	1.06 (0.96-1.17)	0.225		1.10 (0.98-1.23)	0.096
Private medical insurance	0.74 (0.68-0.81)	<0.001		0.77 (0.69-0.85)	<0.001

© 2022 Brumbaugh JE et al. *JAMA Network Open.*

	Unadjusted results			Adjusted results[a]	
Characteristics	OR (95% CI)	P-value		OR (95% CI)	P-value
Antenatal steroids	0.58 (0.52-0.65)	<0.001		0.83 (0.72-0.95)	0.008
Male	1.27 (1.16-1.38)	<0.001		1.23 (1.12-1.36)	<0.001
Small for gestational age	2.58 (2.24-2.97)	<0.001		4.04 (3.42-4.78)	<0.001
Severe ICH[c] (grade III or IV)	3.92 (3.55-4.32)	<0.001		3.39 (3.03-3.79)	<0.001
Infection status					
LOM[d]	1.85 (1.32-2.61)	0.001		1.33 (0.89-1.97)	0.161
LOS[e] and no LOM[d]	1.52 (1.40-1.66)	<0.001		1.23 (1.11-1.36)	<0.001
No LOS[e] or LOM[d]	REF			REF	

[a] Odds ratio and p-values are from logistic regression model which included all covariates listed in the table and center.
[b] NDI, neurodevelopmental impairment.
[c] ICH, intracranial hemorrhage.
[d] LOM, late-onset meningitis.
[e] LOS, late-onset sepsis.

eTable 6. Outcomes at 18 to 26 Months' Corrected Age by Late-Onset Meningitis Pathogen Classification

Characteristics	n	CoNS[a] LOM n=47	n	Non-CoNS[a] Bacterial LOM n=70	n	Fungal LOM n=34	P-value[b]
NDI[c] or Death	46	22 (48)	67	43 (64)	33	26 (79)	0.018
NDI[c]	36	12 (33)	39	15 (38)	20	13 (65)	0.059
Death	47	10 (21)	70	28 (40)	34	13 (38)	0.091

[a] CoNS, coagulase negative *Staphylococcus*.
[b] P-values were calculated from chi-square tests.
[c] NDI, neurodevelopmental impairment.

© 2022 Brumbaugh JE et al. *JAMA Network Open.*

eFigure 1. Incidence of Detected Late-Onset Meningitis by Center (2003-2017)

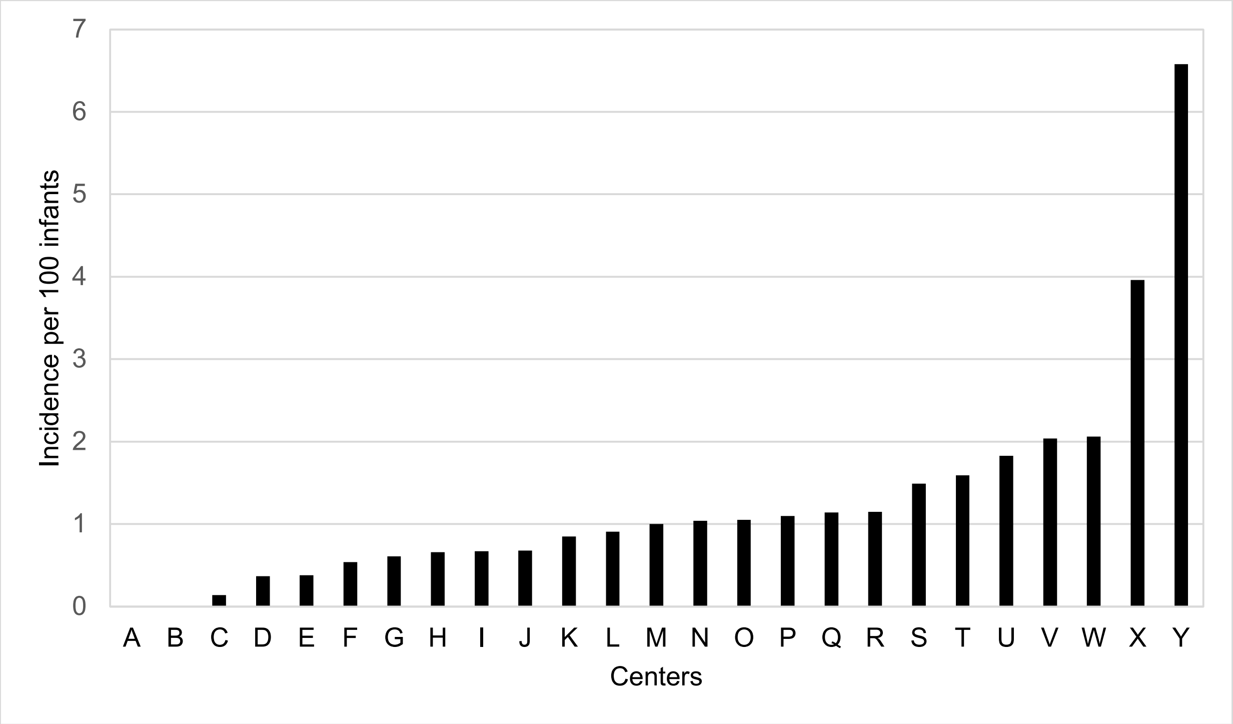

© 2022 Brumbaugh JE et al. *JAMA Network Open.*

eFigure 2. Incidence of Lumbar Puncture (LP) in Late-Onset Sepsis Evaluations by Center, and Percentage of Infants Diagnosed with Late-Onset Meningitis (2011-2017)[a]

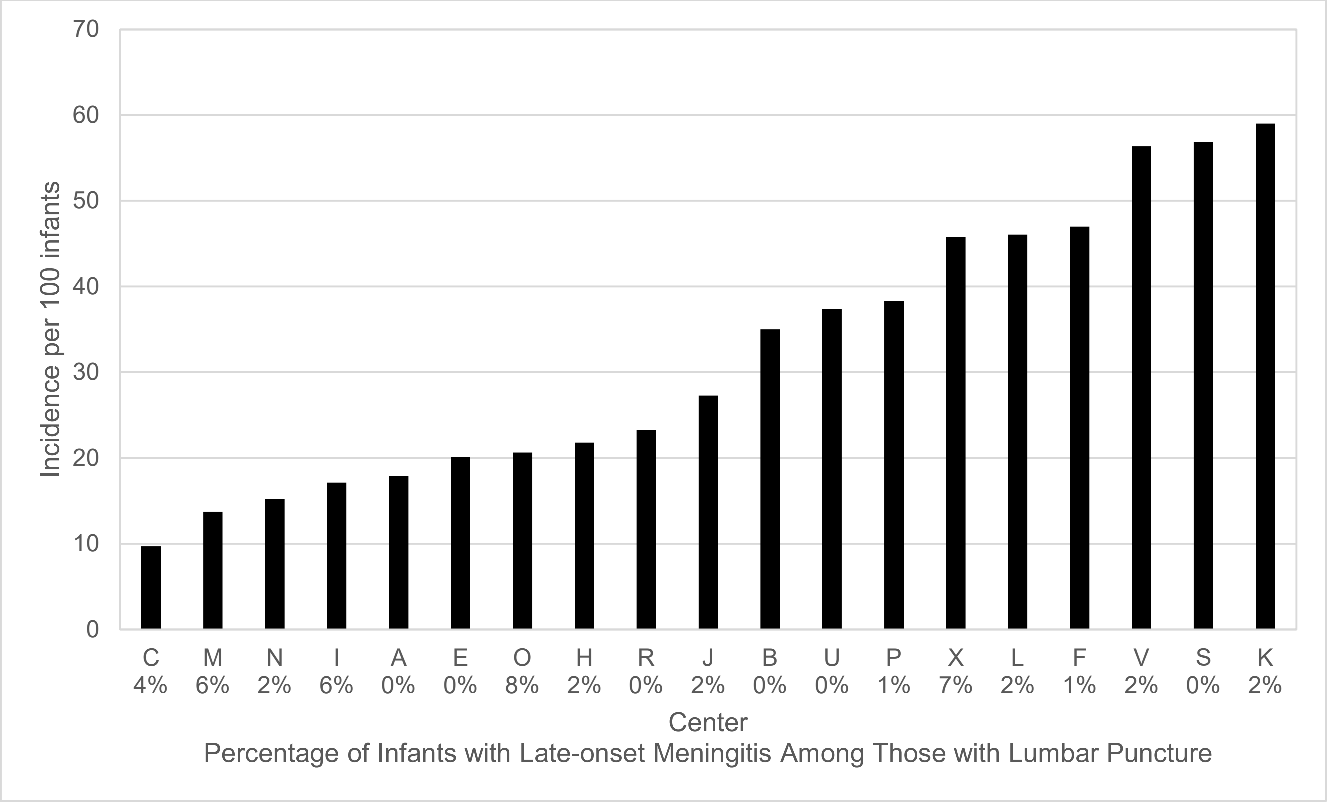

[a]There were six fewer centers in the NRN for the period 2011-2017 compared to the full period 2003-2017. The centers are represented by the same letter in eFigures 1-3.

© 2022 Brumbaugh JE et al. *JAMA Network Open.*

eFigure 3. Incidence of Lumbar Puncture Among Infants with Culture-confirmed Late-Onset Sepsis by Center (2011-2017)[a]

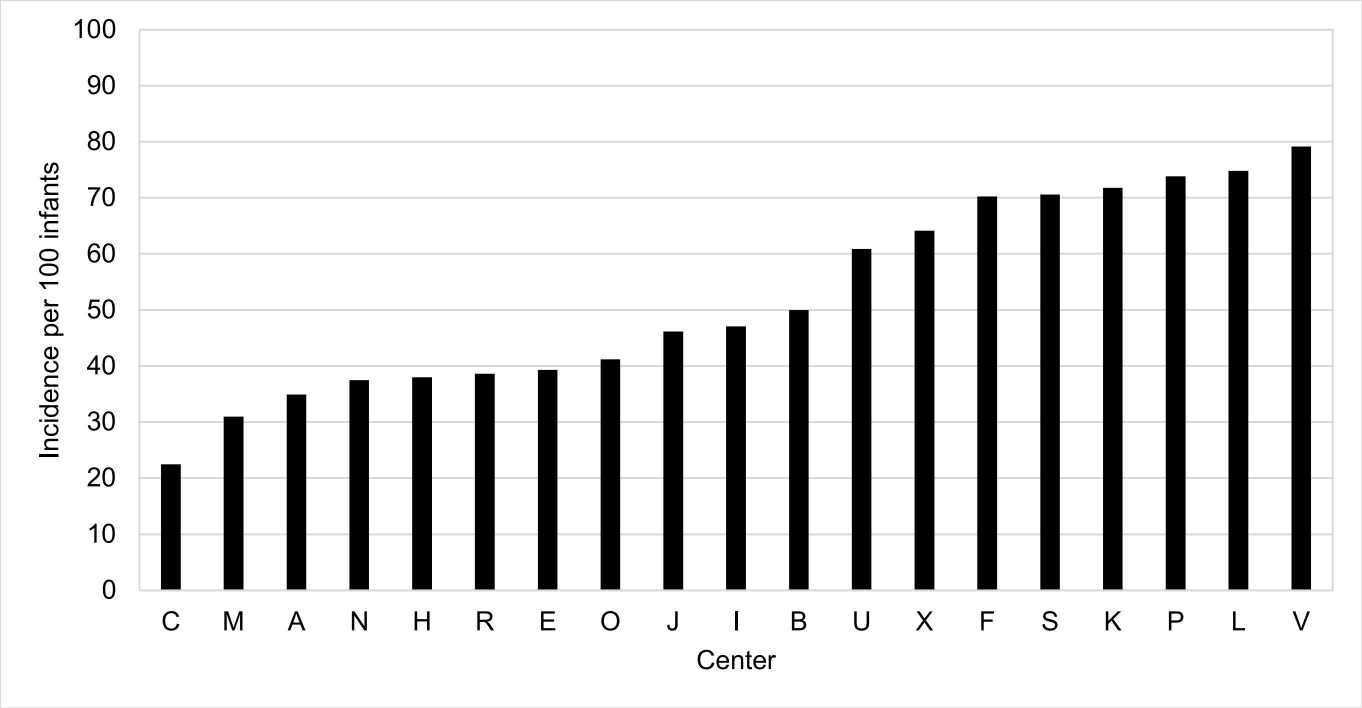

[a]There were six fewer centers in the NRN for the period 2011-2017 compared to the full period 2003-2017. The centers are represented by the same letter in eFigures 1-3.

© 2022 Brumbaugh JE et al. *JAMA Network Open.*
