# Supplementary material for: Incidence of and Neurodevelopmental Outcomes After Late-Onset Meningitis Among Children Born Extremely Preterm
Source: JAMA Netw Open. 2022 Dec 8;5(12):e2245826. doi: 10.1001/jamanetworkopen.2022.45826 (PMC9856224; doi:10.1001/jamanetworkopen.2022.45826)
Supplement: Supplement 2. — Nonauthor Collaborators [file jamanetwopen-e2245826-s002.pdf]

Supplemental Online Content: Nonauthor Collaborators
*First name, last name, and suffix (if applicable) are required and will appear in PubMed.

***Group Name(s): Eunice Kennedy Shriver National Institute of Child Health and Human Development Neonatal Research Network**

*First Name and Middle Initial(s)	*Last Name	*Suffix (eg, Jr, III)	Academic Degrees	Institution	Location (city, state/province, country)	Role or Contribution, eg, chair, principal investigator	Group (if more than 1 Group listed in the byline) and/or Subgroup (eg, Steering Committee)
Namasivayam	Ambalavanan		MD	Division of Neonatology, University c	Birmingham, AL, USA	Co-Principal Investigat	NICHD Neonatal Research Network
Kirstin J.	Bailey		PhD	Division of Neonatology, University c	Birmingham, AL, USA	Follow-up Examiner	NICHD Neonatal Research Network
Fred J.	Biasini		PhD	Division of Neonatology, University c	Birmingham, AL, USA	Follow-up Examiner	NICHD Neonatal Research Network
Waldemar A.	Carlo		MD	Division of Neonatology, University c	Birmingham, AL, USA	Principal Investigator	NICHD Neonatal Research Network
Stephanie A.	Chopko		PhD	Division of Neonatology, University c	Birmingham, AL, USA	Follow-up Examiner	NICHD Neonatal Research Network
Monica V.	Collins		RN BSN Ma	Division of Neonatology, University c	Birmingham, AL, USA	Research Coordinator	NICHD Neonatal Research Network
Shirley S.	Cosby		RN BSN	Division of Neonatology, University c	Birmingham, AL, USA	Research Coordinator	NICHD Neonatal Research Network
Kristen C.	Johnston		MSN CRNP	Division of Neonatology, University c	Birmingham, AL, USA	Follow-up Examiner	NICHD Neonatal Research Network
Mary Beth	Moses		PT MS PCS	Division of Neonatology, University c	Birmingham, AL, USA	Follow-up Examiner	NICHD Neonatal Research Network
Kathleen G.	Nelson		MD	Division of Neonatology, University c	Birmingham, AL, USA	Follow-up Examiner	NICHD Neonatal Research Network
Cryshelle S.	Patterson		PhD	Division of Neonatology, University c	Birmingham, AL, USA	Follow-up Examiner	NICHD Neonatal Research Network
Myriam	Peralta-Carcelen		MD MPH	Division of Neonatology, University c	Birmingham, AL, USA	Follow-up Principal Inv	NICHD Neonatal Research Network
Vivien A.	Phillips		RN BSN	Division of Neonatology, University c	Birmingham, AL, USA	Follow-up Coordinator	NICHD Neonatal Research Network
Julie	Preskitt		PhD MSOT	Division of Neonatology, University c	Birmingham, AL, USA	Follow-up Examiner	NICHD Neonatal Research Network
Richard V.	Rector		PhD	Division of Neonatology, University c	Birmingham, AL, USA	Follow-up Examiner	NICHD Neonatal Research Network
Sally	Whitley		MA OTR-L I	Division of Neonatology, University c	Birmingham, AL, USA	Follow-up Examiner	NICHD Neonatal Research Network
Barbara	Alksninis		RNC PNP	Department of Pediatrics, Women &	Providence, RI, USA	Follow-up Examiner	NICHD Neonatal Research Network
Carmena	Bishop			Department of Pediatrics, Women &	Providence, RI, USA	Follow-up Coordinator	NICHD Neonatal Research Network
Robert T.	Burke		MD MPH	Department of Pediatrics, Women &	Providence, RI, USA		NICHD Neonatal Research Network
Melinda	Caskey		MD	Department of Pediatrics, Women &	Providence, RI, USA	Follow-up Examiner	NICHD Neonatal Research Network
Angelita M.	Hensman		PhD RNC-N	Department of Pediatrics, Women &	Providence, RI, USA	Research Coordinator	NICHD Neonatal Research Network
Laurie	Hoffman		MD	Department of Pediatrics, Women &	Providence, RI, USA		NICHD Neonatal Research Network
Katharine	Johnson		MD	Department of Pediatrics, Women &	Providence, RI, USA	Follow-up Examiner	NICHD Neonatal Research Network
Martin	Keszler		MD	Department of Pediatrics, Women &	Providence, RI, USA	Co-Principal Investigat	NICHD Neonatal Research Network
Mary Lenore	Keszler		MD	Department of Pediatrics, Women &	Providence, RI, USA	Follow-up Examiner	NICHD Neonatal Research Network
Andrea M.	Knoll			Department of Pediatrics, Women &	Providence, RI, USA		NICHD Neonatal Research Network
Vita	Lamberson		MD	Department of Pediatrics, Women &	Providence, RI, USA		NICHD Neonatal Research Network
Abbot R.	Laptook		MD	Department of Pediatrics, Women &	Providence, RI, USA	Principal Investigator	NICHD Neonatal Research Network
Theresa M.	Leach		MEd CAES	Department of Pediatrics, Women &	Providence, RI, USA	Follow-up Examiner	NICHD Neonatal Research Network
Emilee	Little		RN BSN	Department of Pediatrics, Women &	Providence, RI, USA		NICHD Neonatal Research Network
Elizabeth C.	McGowan		MD	Department of Pediatrics, Women &	Providence, RI, USA	Follow-up Examiner	NICHD Neonatal Research Network

Supplemental Online Content: Nonauthor Collaborators
*First name, last name, and suffix (if applicable) are required and will appear in PubMed.

*First Name and Middle Initial(s)	*Last Name	*Suffix (eg, Jr, III)	Academic Degrees	Institution	Location (city, state/province, country)	Role or Contribution, eg, chair, principal investigator	Group (if more than 1 Group listed in the byline) and/or Subgroup (eg, Steering Committee)
William	Oh		MD	Department of Pediatrics, Women &	Providence, RI, USA	Steering Committee Ch	NICHD Neonatal Research Network
Lucille	St. Pierre		BS	Department of Pediatrics, Women &	Providence, RI, USA	Research Assistant	NICHD Neonatal Research Network
Bonnie E.	Stephens		MD	Department of Pediatrics, Women &	Providence, RI, USA	Follow-up Coordinator	NICHD Neonatal Research Network
Suzy	Ventura			Department of Pediatrics, Women &	Providence, RI, USA		NICHD Neonatal Research Network
Elisa	Vieira		RN BSN	Department of Pediatrics, Women &	Providence, RI, USA	Research Nurse	NICHD Neonatal Research Network
Betty R.	Vohr		MD	Department of Pediatrics, Women &	Providence, RI, USA	Follow-up Principal Inv	NICHD Neonatal Research Network
Victoria E.	Watson		MS CAS	Department of Pediatrics, Women &	Providence, RI, USA	Follow-up Examiner	NICHD Neonatal Research Network
Kelly R.	Coleman		PsyD	Department of Pediatrics, University	Buffalo, NY, USA	Follow-up Examiner	NICHD Neonatal Research Network
Stephanie	Guilford		BS	Department of Pediatrics, University	Buffalo, NY, USA	Research Coordinator	NICHD Neonatal Research Network
Michelle E.	Hartley-McAndre		MD	Department of Pediatrics, University	Buffalo, NY, USA	Follow-up Principal Inv	NICHD Neonatal Research Network
Satyan	Lakshminrusimha		MD	Department of Pediatrics, University	Buffalo, NY, USA	Co-Principal Investigat	NICHD Neonatal Research Network
Emily	Li		BA	Department of Pediatrics, University	Buffalo, NY, USA	Research Nurse	NICHD Neonatal Research Network
Anne Marie	Reynolds		MD MPH	Department of Pediatrics, University	Buffalo, NY, USA	Site Investigator	NICHD Neonatal Research Network
Michael G.	Sacilowski		MAT CCRC	Department of Pediatrics, University	Buffalo, NY, USA	Follow-up Coordinator	NICHD Neonatal Research Network
Ashley	Williams		MS Ed	Department of Pediatrics, University	Buffalo, NY, USA	Research Coordinator	NICHD Neonatal Research Network
William A.	Zorn		PhD	Department of Pediatrics, University	Buffalo, NY, USA		NICHD Neonatal Research Network
Monika	Bhola		MD	Department of Pediatrics, Rainbow B	Cleveland, OH, USA	Follow-up Examiner	NICHD Neonatal Research Network
Avroy A.	Fanaroff		MD	Department of Pediatrics, Rainbow B	Cleveland, OH, USA	Principal Investigator	NICHD Neonatal Research Network
Harriet G.	Friedman		MA	Department of Pediatrics, Rainbow B	Cleveland, OH, USA	Follow-up Examiner	NICHD Neonatal Research Network
Anna Maria	Hibbs		MD MSCE	Department of Pediatrics, Rainbow B	Cleveland, OH, USA	Principal Investigator	NICHD Neonatal Research Network
Nancy S.	Newman		RN	Department of Pediatrics, Rainbow B	Cleveland, OH, USA	Research Coordinator	NICHD Neonatal Research Network
Allison H.	Payne		MD MSCR	Department of Pediatrics, Rainbow B	Cleveland, OH, USA	Follow-up Examiner	NICHD Neonatal Research Network
Bonnie S.	Siner		RN	Department of Pediatrics, Rainbow B	Cleveland, OH, USA	Follow-up Coordinator	NICHD Neonatal Research Network
Michele C.	Walsh		MD MS	Department of Pediatrics, Rainbow B	Cleveland, OH, USA	Co-Principal Investigat	NICHD Neonatal Research Network
Deanne E.	Wilson-Costello		MD	Department of Pediatrics, Rainbow B	Cleveland, OH, USA	Follow-up Principal Inv	NICHD Neonatal Research Network
Gulgun	Yalcinkaya		MD	Department of Pediatrics, Rainbow B	Cleveland, OH, USA	Follow-up Examiner	NICHD Neonatal Research Network
Michael S.	Caplan		MD	Pritzker School of Medicine, Universi	Chicago, IL, USA	Steering Committee Ch	NICHD Neonatal Research Network
Barbara	Alexander		RN	Cincinnati Children’s Hospital Medica	Cincinnati, OH, USA	Research Coordinator	NICHD Neonatal Research Network
Traci	Beiersdorfer		RN BSN	Cincinnati Children’s Hospital Medica	Cincinnati, OH, USA	Research Nurse	NICHD Neonatal Research Network
Kate	Bridges		MD	Cincinnati Children’s Hospital Medica	Cincinnati, OH, USA		NICHD Neonatal Research Network
Tanya E.	Cahill		MD	Cincinnati Children’s Hospital Medica	Cincinnati, OH, USA	Follow-up Examiner	NICHD Neonatal Research Network
Edward F.	Donovan		MD	Cincinnati Children’s Hospital Medica	Cincinnati, OH, USA	Principal Investigator	NICHD Neonatal Research Network
Juanita	Dudley		RN BSN	Cincinnati Children’s Hospital Medica	Cincinnati, OH, USA		NICHD Neonatal Research Network

Supplemental Online Content: Nonauthor Collaborators
*First name, last name, and suffix (if applicable) are required and will appear in PubMed.

***First Name and Middle Initial(s)**	***Last Name**	***Suffix (eg, Jr, III)**	Academic Degrees	Institution	Location (city, state/province, country)	Role or Contribution, eg, chair, principal investigator	Group (if more than 1 Group listed in the byline) and/or Subgroup (eg, Steering Committee)
Estelle E.	Fischer		MHSA MBA	Cincinnati Children's Hospital Medica	Cincinnati, OH, USA		NICHD Neonatal Research Network
Teresa L.	Gratton		PA	Cincinnati Children's Hospital Medica	Cincinnati, OH, USA	Follow-up Coordinator	NICHD Neonatal Research Network
Cathy	Grisby		BSN CCRC	Cincinnati Children's Hospital Medica	Cincinnati, OH, USA	Research Coordinator	NICHD Neonatal Research Network
Devan	Hayes		BS	Cincinnati Children's Hospital Medica	Cincinnati, OH, USA		NICHD Neonatal Research Network
Jody	Hessling		MSN RN	Cincinnati Children's Hospital Medica	Cincinnati, OH, USA	Research Coordinator	NICHD Neonatal Research Network
Lenora Denise	Jackson		CRC	Cincinnati Children's Hospital Medica	Cincinnati, OH, USA		NICHD Neonatal Research Network
Alan H.	Jobe		MD PhD	University of Cincinnati	Cincinnati, OH, USA	Steering Committee Ch	NICHD Neonatal Research Network
Suhas G.	Kallapur		MD	Cincinnati Children's Hospital Medica	Cincinnati, OH, USA	Co-Principal Investigat	NICHD Neonatal Research Network
Kristin	Kirker		CRC	Cincinnati Children's Hospital Medica	Cincinnati, OH, USA	Research Nurse	NICHD Neonatal Research Network
Stephanie L.	Merhar		MD MS	Cincinnati Children's Hospital Medica	Cincinnati, OH, USA	Follow-up Principal Inv	NICHD Neonatal Research Network
Holly L.	Mincey		MS RN BSN	Cincinnati Children's Hospital Medica	Cincinnati, OH, USA	Research Coordinator	NICHD Neonatal Research Network
Greg	Muthig		BA	Cincinnati Children's Hospital Medica	Cincinnati, OH, USA	Research Nurse	NICHD Neonatal Research Network
Brenda B.	Poindexter		MD MS	Cincinnati Children's Hospital Medica	Cincinnati, OH, USA	Co-Principal Investigat	NICHD Neonatal Research Network
Kurt	Schibler		MD	Cincinnati Children's Hospital Medica	Cincinnati, OH, USA	Principal Investigator	NICHD Neonatal Research Network
Sara	Stacey		BA	Cincinnati Children's Hospital Medica	Cincinnati, OH, USA		NICHD Neonatal Research Network
Jean J.	Steichen		MD	Cincinnati Children's Hospital Medica	Cincinnati, OH, USA	Follow-up Medical Dire	NICHD Neonatal Research Network
Stacey	Tepe		BS	Cincinnati Children's Hospital Medica	Cincinnati, OH, USA		NICHD Neonatal Research Network
Julia	Thompson		RN BSN	Cincinnati Children's Hospital Medica	Cincinnati, OH, USA	Research Nurse	NICHD Neonatal Research Network
Marcia	Worley Mersmar		BS RN CCR(	Cincinnati Children's Hospital Medica	Cincinnati, OH, USA	Research Coordinator	NICHD Neonatal Research Network
Sandra	Wuertz		RN BSN CC	Cincinnati Children's Hospital Medica	Cincinnati, OH, USA	Research Nurse	NICHD Neonatal Research Network
Kimberly	Yolton		PhD	Cincinnati Children's Hospital Medica	Cincinnati, OH, USA	Follow-up Principal Inv	NICHD Neonatal Research Network
Richard A.	Polin		MD	Division of Neonatology, College of P	New York, NY, USA	Steering Committee Ch	NICHD Neonatal Research Network
Sally S.	Adams		MS RN CPN	Department of Pediatrics, University	Dallas, TX, USA	Follow-up Examiner	NICHD Neonatal Research Network
Luc P.	Brion		MD	Department of Pediatrics, University	Dallas, TX, USA	Co-Principal Investigat	NICHD Neonatal Research Network
Lijun	Chen		PhD RN	Department of Pediatrics, University	Dallas, TX, USA	Research Coordinator	NICHD Neonatal Research Network
Maria M.	De Leon		RN BSN	Department of Pediatrics, University	Dallas, TX, USA	Research Nurse	NICHD Neonatal Research Network
Frances	Eubanks		RN BSN	Department of Pediatrics, University	Dallas, TX, USA	Research Nurse	NICHD Neonatal Research Network
Alicia	Guzman			Department of Pediatrics, University	Dallas, TX, USA	Research Coordinator	NICHD Neonatal Research Network
Gaynelle	Hensley		RN	Department of Pediatrics, University	Dallas, TX, USA	Research Coordinator	NICHD Neonatal Research Network
Elizabeth T.	Heyne		MS MA PA-	Department of Pediatrics, University	Dallas, TX, USA	Follow-up Examiner	NICHD Neonatal Research Network
Roy J.	Heyne		MD	Department of Pediatrics, University	Dallas, TX, USA	Follow-up Principal Inv	NICHD Neonatal Research Network
Jackie F.	Hickman		RN	Department of Pediatrics, University	Dallas, TX, USA	Follow-up Coordinator	NICHD Neonatal Research Network
Lizette E.	Lee		RN	Department of Pediatrics, University	Dallas, TX, USA	Research Nurse	NICHD Neonatal Research Network

***First Name and Middle Initial(s)**	***Last Name**	***Suffix (eg, Jr, III)**	Academic Degrees	Institution	Location (city, state/province, country)	Role or Contribution, eg, chair, principal investigator	Group (if more than 1 Group listed in the byline) and/or Subgroup (eg, Steering Committee)
Melissa H.	Leps		RN	Department of Pediatrics, University	Dallas, TX, USA	Research Coordinator	NICHD Neonatal Research Network
Linda A.	Madden		RN BSN CPI	Department of Pediatrics, University	Dallas, TX, USA	Follow-up Examiner	NICHD Neonatal Research Network
E. Rebecca	McDougald		MSN APRN	Department of Pediatrics, University	Dallas, TX, USA		NICHD Neonatal Research Network
Nancy A.	Miller		RN	Department of Pediatrics, University	Dallas, TX, USA	Research Coordinator	NICHD Neonatal Research Network
Janet S.	Morgan		RN	Department of Pediatrics, University	Dallas, TX, USA	Follow-up Coordinator	NICHD Neonatal Research Network
Lara	Pavageau		MD	Department of Pediatrics, University	Dallas, TX, USA	Site Investigator	NICHD Neonatal Research Network
Charles R.	Rosenfeld		MD	Department of Pediatrics, University	Dallas, TX, USA	Principal Investigator	NICHD Neonatal Research Network
Walid A.	Salhab		MD	Department of Pediatrics, University	Dallas, TX, USA	Co-Principal Investigat	NICHD Neonatal Research Network
Pablo J.	Sánchez		MD	Department of Pediatrics, University	Dallas, TX, USA		NICHD Neonatal Research Network
Pollieanna	Sepulveda		RN BSN	Department of Pediatrics, University	Dallas, TX, USA	Research Nurse	NICHD Neonatal Research Network
Catherine	Twell Boatman		MS CIMI	Department of Pediatrics, University	Dallas, TX, USA	Follow-up Examiner	NICHD Neonatal Research Network
Diana M.	Vasil		MSN RNC-N	Department of Pediatrics, University	Dallas, TX, USA	Research Coordinator	NICHD Neonatal Research Network
Jillian	Waterbury		DNP RN CP	Department of Pediatrics, University	Dallas, TX, USA		NICHD Neonatal Research Network
Myra H.	Wyckoff		MD	Department of Pediatrics, University	Dallas, TX, USA	Principal Investigator	NICHD Neonatal Research Network
Patricia L.	Ashley		MD PhD	Department of Pediatrics, Duke Univ	Durham, NC, USA	Follow-up Examiner	NICHD Neonatal Research Network
Kathy J.	Auten		MSHS	Department of Pediatrics, Duke Univ	Durham, NC, USA	Research Coordinator	NICHD Neonatal Research Network
C. Michael	Cotten		MD MHS	Department of Pediatrics, Duke Univ	Durham, NC, USA	Co-Principal Investigat	NICHD Neonatal Research Network
Joanne	Finkle		RN JD	Department of Pediatrics, Duke Univ	Durham, NC, USA	Research Coordinator	NICHD Neonatal Research Network
Kimberley A.	Fisher		PhD FNP-B	Department of Pediatrics, Duke Univ	Durham, NC, USA	Research Coordinator	NICHD Neonatal Research Network
Ronald N.	Goldberg		MD	Department of Pediatrics, Duke Univ	Durham, NC, USA	Co-Principal Investigat	NICHD Neonatal Research Network
Ricki F.	Goldstein		MD	Department of Pediatrics, Duke Univ	Durham, NC, USA	Follow-up Principal Inv	NICHD Neonatal Research Network
Sandra	Grimes		RN BSN	Department of Pediatrics, Duke Univ	Durham, NC, USA	Follow-up Coordinator	NICHD Neonatal Research Network
Kathryn E.	Gustafson		PhD	Department of Pediatrics, Duke Univ	Durham, NC, USA	Follow-up Examiner	NICHD Neonatal Research Network
Melody B.	Lohmeyer		RN MSN	Department of Pediatrics, Duke Univ	Durham, NC, USA	Follow-up Coordinator	NICHD Neonatal Research Network
William F.	Malcolm		MD	Department of Pediatrics, Duke Univ	Durham, NC, USA	Follow-up Examiner	NICHD Neonatal Research Network
Ira	Adams-Chapmar		MD MPH	Department of Pediatrics, Emory Uni	Atlanta, GA, USA	Follow-up Principal Inv	NICHD Neonatal Research Network
Ann	Blackwelder		MN RN	Department of Pediatrics, Emory Uni	Atlanta, GA, USA	Site Investigator	NICHD Neonatal Research Network
Diane I.	Bottcher		RN MSN	Department of Pediatrics, Emory Uni	Atlanta, GA, USA	Research Nurse	NICHD Neonatal Research Network
David P.	Carlton		MD	Department of Pediatrics, Emory Uni	Atlanta, GA, USA	Principal Investigator	NICHD Neonatal Research Network
Sheena L.	Carter		PhD	Department of Pediatrics, Emory Uni	Atlanta, GA, USA	Follow-up Examiner	NICHD Neonatal Research Network
Ellen C.	Hale		RN BS CCR(	Department of Pediatrics, Emory Uni	Atlanta, GA, USA	Research Coordinator	NICHD Neonatal Research Network
Salathiel	Kendrick-Allwoo		MD	Department of Pediatrics, Emory Uni	Atlanta, GA, USA		NICHD Neonatal Research Network
Judith	Laursen		RN	Department of Pediatrics, Emory Uni	Atlanta, GA, USA		NICHD Neonatal Research Network

Supplemental Online Content: Nonauthor Collaborators
*First name, last name, and suffix (if applicable) are required and will appear in PubMed.

*First Name and Middle Initial(s)	*Last Name	*Suffix (eg, Jr, III)	Academic Degrees	Institution	Location (city, state/province, country)	Role or Contribution, eg, chair, principal investigator	Group (if more than 1 Group listed in the byline) and/or Subgroup (eg, Steering Committee)
Yvonne C.	Loggins		RN BSN	Department of Pediatrics, Emory Uni	Atlanta, GA, USA	Research Coordinator	NICHD Neonatal Research Network
Colleen	Mackie		BS RT	Department of Pediatrics, Emory Uni	Atlanta, GA, USA	Research Nurse	NICHD Neonatal Research Network
Maureen	Mulligan LaRossa		RN	Department of Pediatrics, Emory Uni	Atlanta, GA, USA	Follow-up Examiner	NICHD Neonatal Research Network
Ravi M.	Patel		MD MSc	Department of Pediatrics, Emory Uni	Atlanta, GA, USA	Co-Principal Investigat	NICHD Neonatal Research Network
Amy	Sanders		PsyD	Department of Pediatrics, Emory Uni	Atlanta, GA, USA		NICHD Neonatal Research Network
Irma	Seabrook		RRT	Department of Pediatrics, Emory Uni	Atlanta, GA, USA		NICHD Neonatal Research Network
Gloria V.	Smikle		PNP MSN	Department of Pediatrics, Emory Uni	Atlanta, GA, USA	Follow-up Examiner	NICHD Neonatal Research Network
Lynn	Wineski		NNP	Department of Pediatrics, Emory Uni	Atlanta, GA, USA	Follow-up Examiner	NICHD Neonatal Research Network
Esther G.	Akpa		RN BSN	Department of Pediatrics, McGovern	Houston, TX, USA	Research Coordinator	NICHD Neonatal Research Network
Nora I.	Alaniz		BS	Department of Pediatrics, McGovern	Houston, TX, USA		NICHD Neonatal Research Network
Elizabeth	Allain		PhD	Department of Pediatrics, McGovern	Houston, TX, USA	Follow-up Examiner	NICHD Neonatal Research Network
Julie	Arldt-McAlister		MSN APRN	Department of Pediatrics, McGovern	Houston, TX, USA	Research Nurse	NICHD Neonatal Research Network
Pamela J.	Bradt		MD MPH	Department of Pediatrics, McGovern	Houston, TX, USA	Follow-up Principal Inv	NICHD Neonatal Research Network
Katrina	Burson		RN BSN	Department of Pediatrics, McGovern	Houston, TX, USA	Research Nurse	NICHD Neonatal Research Network
Allison G.	Dempsey		PhD	Department of Pediatrics, McGovern	Houston, TX, USA	Follow-up Examiner	NICHD Neonatal Research Network
Susan E.	Dieterich		PhD	Department of Pediatrics, McGovern	Houston, TX, USA	Follow-up Examiner	NICHD Neonatal Research Network
Andrea F.	Duncan		MD MSClin	Department of Pediatrics, McGovern	Houston, TX, USA	Follow-up Principal Inv	NICHD Neonatal Research Network
Elizabeth	Eason		MD	Department of Pediatrics, McGovern	Houston, TX, USA	Site Investigator	NICHD Neonatal Research Network
Patricia W.	Evans		MD	Department of Pediatrics, McGovern	Houston, TX, USA	Follow-up Principal Inv	NICHD Neonatal Research Network
Beverly	Foley Harris		RN BSN	Department of Pediatrics, McGovern	Houston, TX, USA	Research Nurse	NICHD Neonatal Research Network
Claudia I.	Franco		RNC MSN	Department of Pediatrics, McGovern	Houston, TX, USA	Research Coordinator	NICHD Neonatal Research Network
Carmen	Garcia		RN BSN	Department of Pediatrics, McGovern	Houston, TX, USA	Research Nurse	NICHD Neonatal Research Network
Charles E.	Green		PhD	Department of Pediatrics, McGovern	Houston, TX, USA	Follow-up Examiner	NICHD Neonatal Research Network
Donna J.	Hall		RN	Department of Pediatrics, McGovern	Houston, TX, USA		NICHD Neonatal Research Network
Margarita	Jimenez		MD MPH	Department of Pediatrics, McGovern	Houston, TX, USA	Follow-up Examiner	NICHD Neonatal Research Network
Janice	John		CPNP	Department of Pediatrics, McGovern	Houston, TX, USA		NICHD Neonatal Research Network
Patrick M.	Jones		MD MA	Department of Pediatrics, McGovern	Houston, TX, USA		NICHD Neonatal Research Network
Kathleen A.	Kennedy		MD MPH	Department of Pediatrics, McGovern	Houston, TX, USA	Principal Investigator	NICHD Neonatal Research Network
Amir M.	Khan		MD	Department of Pediatrics, McGovern	Houston, TX, USA	Site Investigator	NICHD Neonatal Research Network
Saba	Khan Siddiki		MD	Department of Pediatrics, McGovern	Houston, TX, USA		NICHD Neonatal Research Network
Layne	Lillie		RN BSN	Department of Pediatrics, McGovern	Houston, TX, USA		NICHD Neonatal Research Network
Anna E.	Lis		RN BSN	Department of Pediatrics, McGovern	Houston, TX, USA		NICHD Neonatal Research Network
Terri L.	Major-Kincade		MD MPH	Department of Pediatrics, McGovern	Houston, TX, USA	Follow-up Examiner	NICHD Neonatal Research Network

Supplemental Online Content: Nonauthor Collaborators
*First name, last name, and suffix (if applicable) are required and will appear in PubMed.

*First Name and Middle Initial(s)	*Last Name	*Suffix (eg, Jr, III)	Academic Degrees	Institution	Location (city, state/province, country)	Role or Contribution, eg, chair, principal investigator	Group (if more than 1 Group listed in the byline) and/or Subgroup (eg, Steering Committee)
Karen	Martin		RN	Department of Pediatrics, McGovern	Houston, TX, USA	Research Nurse	NICHD Neonatal Research Network
Sara C.	Martin		RN BSN	Department of Pediatrics, McGovern	Houston, TX, USA	Research Nurse	NICHD Neonatal Research Network
Georgia Elaine	McDavid		RN	Department of Pediatrics, McGovern	Houston, TX, USA	Research Coordinator	NICHD Neonatal Research Network
Shannon L.	McKee		EdS	Department of Pediatrics, McGovern	Houston, TX, USA		NICHD Neonatal Research Network
Brenda H.	Morris		MD	Department of Pediatrics, McGovern	Houston, TX, USA	Study Investigator	NICHD Neonatal Research Network
Patricia Ann	Orekoya		RN BSN	Department of Pediatrics, McGovern	Houston, TX, USA	Research Coordinator	NICHD Neonatal Research Network
Claudia	Pedrozza		PhD	Department of Pediatrics, McGovern	Houston, TX, USA		NICHD Neonatal Research Network
Carrie M.	Perez		MA	Department of Pediatrics, McGovern	Houston, TX, USA		NICHD Neonatal Research Network
Patti L.	Pierce Tate		RCP	Department of Pediatrics, McGovern	Houston, TX, USA	Research Nurse	NICHD Neonatal Research Network
Stacey	Reddoch		BA	Department of Pediatrics, McGovern	Houston, TX, USA		NICHD Neonatal Research Network
Shawna	Rodgers		RN BSN	Department of Pediatrics, McGovern	Houston, TX, USA		NICHD Neonatal Research Network
Maegan C.	Simmons		RN	Department of Pediatrics, McGovern	Houston, TX, USA	Research Nurse	NICHD Neonatal Research Network
Daniel K.	Sperry		RN	Department of Pediatrics, McGovern	Houston, TX, USA	Research Nurse	NICHD Neonatal Research Network
Emily K.	Stephens		BSN RNC-N	Department of Pediatrics, McGovern	Houston, TX, USA	Research Coordinator	NICHD Neonatal Research Network
Jon E.	Tyson		MD MPH	Department of Pediatrics, McGovern	Houston, TX, USA	Principal Investigator	NICHD Neonatal Research Network
Laura L.	Whitely		MD	Department of Pediatrics, McGovern	Houston, TX, USA	Follow-up Examiner	NICHD Neonatal Research Network
Sharon L.	Wright		MT (ASCP)	Department of Pediatrics, McGovern	Houston, TX, USA		NICHD Neonatal Research Network
Anna M.	Dusick		MD	Department of Pediatrics, Indiana Ur	Indianapolis, IN, USA	Follow-up Principal Inv	NICHD Neonatal Research Network
Susan	Gunn		NNP-BC CC	Department of Pediatrics, Indiana Ur	Indianapolis, IN, USA	Research Nurse	NICHD Neonatal Research Network
Faithe	Hamer		BS	Department of Pediatrics, Indiana Ur	Indianapolis, IN, USA	Follow-up Coordinator	NICHD Neonatal Research Network
Dianne E.	Herron		RN CCRC	Department of Pediatrics, Indiana Ur	Indianapolis, IN, USA	Research Coordinator	NICHD Neonatal Research Network
Abbey C.	Hines		PsyD	Department of Pediatrics, Indiana Ur	Indianapolis, IN, USA	Follow-up Examiner	NICHD Neonatal Research Network
Jeffery	Joyce		CCRC	Department of Pediatrics, Indiana Ur	Indianapolis, IN, USA	Research Coordinator (	NICHD Neonatal Research Network
James A.	Lemons		MD	Department of Pediatrics, Indiana Ur	Indianapolis, IN, USA	Co-Principal Investigat	NICHD Neonatal Research Network
Carolyn	Lytle		MD MPH	Department of Pediatrics, Indiana Ur	Indianapolis, IN, USA	Follow-up Examiner	NICHD Neonatal Research Network
Lucy C.	Miller		RN BSN CC	Department of Pediatrics, Indiana Ur	Indianapolis, IN, USA	Research Coordinator	NICHD Neonatal Research Network
Heike M.	Minnich		PsyD HSPP	Department of Pediatrics, Indiana Ur	Indianapolis, IN, USA	Follow-up Examiner	NICHD Neonatal Research Network
Lu-Ann	Papile		MD	Department of Pediatrics, Indiana Ur	Indianapolis, IN, USA	Follow-up Principal Inv	NICHD Neonatal Research Network
Brenda B.	Poindexter		MD MS	Department of Pediatrics, Indiana Ur	Indianapolis, IN, USA	Co-Principal Investigat	NICHD Neonatal Research Network
Leslie	Richard		RN	Department of Pediatrics, Indiana Ur	Indianapolis, IN, USA	Follow-up Coordinator	NICHD Neonatal Research Network
Lucy C.	Smiley		CCRC	Department of Pediatrics, Indiana Ur	Indianapolis, IN, USA	Research Nurse	NICHD Neonatal Research Network
Gregory M.	Sokol		MD	Department of Pediatrics, Indiana Ur	Indianapolis, IN, USA	Co-Principal Investigat	NICHD Neonatal Research Network
Leslie Dawn	Wilson		BSN CCRC	Department of Pediatrics, Indiana Ur	Indianapolis, IN, USA	Research Coordinator	NICHD Neonatal Research Network

Supplemental Online Content: Nonauthor Collaborators
*First name, last name, and suffix (if applicable) are required and will appear in PubMed.

*First Name and Middle Initial(s)	*Last Name	*Suffix (eg, Jr, III)	Academic Degrees	Institution	Location (city, state/province, country)	Role or Contribution, eg, chair, principal investigator	Group (if more than 1 Group listed in the byline) and/or Subgroup (eg, Steering Committee)	
Michael J.	Acarregui		MD MBA	Department of Pediatrics, University	Iowa City, IA, USA	Follow-up Principal Inv	NICHD Neonatal Research Network	
Tarah T.	Colaizy		MD MPH	Department of Pediatrics, University	Iowa City, IA, USA	Follow-up Principal Inv	NICHD Neonatal Research Network	
John M.	Dagle		MD PhD	Department of Pediatrics, University	Iowa City, IA, USA	Study Investigator	NICHD Neonatal Research Network	
Diane L.	Eastman		RN CPNP M	Department of Pediatrics, University	Iowa City, IA, USA	Follow-up Coordinator	NICHD Neonatal Research Network	
Claire A.	Goeke		RN	Department of Pediatrics, University	Iowa City, IA, USA	Research Nurse	NICHD Neonatal Research Network	
Karen J.	Johnson		RN BSN	Department of Pediatrics, University	Iowa City, IA, USA	Research Coordinator	NICHD Neonatal Research Network	
Jonathan M.	Klein		MD	Department of Pediatrics, University	Iowa City, IA, USA	Study Investigator	NICHD Neonatal Research Network	
Nancy J.	Krutzfield		RN MA	Department of Pediatrics, University	Iowa City, IA, USA	Research Nurse	NICHD Neonatal Research Network	
Mendi L.	Schmelzel		MSN RN	Department of Pediatrics, University	Iowa City, IA, USA	Research Nurse	NICHD Neonatal Research Network	
Jacky R.	Walker		RN	Department of Pediatrics, University	Iowa City, IA, USA	Research Nurse	NICHD Neonatal Research Network	
John A.	Widness		MD	Department of Pediatrics, University	Iowa City, IA, USA	Co-Principal Investigat	NICHD Neonatal Research Network	
Donia B.	Bass		MS ARNP N	Center for Research, Education, and	Des Moines, IA, USA	Research Coordinator	NICHD Neonatal Research Network	
Dan L.	Ellsbury		MD	Center for Research, Education, and	Des Moines, IA, USA	Site Investigator	NICHD Neonatal Research Network	
Tracy L.	Tud		RN	Center for Research, Education, and	Des Moines, IA, USA	Research Coordinator	NICHD Neonatal Research Network	
Charles R.	Bauer		MD	University of Miami Miller School of	Miami, FL	Follow-up Principal Inv	NICHD Neonatal Research Network	
Maria	Calejo		MEd	University of Miami Miller School of	Miami, FL	Follow-up Examiner	NICHD Neonatal Research Network	
Shahnaz	Duara		MD	University of Miami Miller School of	Miami, FL	Principal Investigator	NICHD Neonatal Research Network	
Ruth	Everett-Thomas		RN MSN	University of Miami Miller School of	Miami, FL	Research Coordinator	NICHD Neonatal Research Network	
Sylvia	Fajardo-Hiriart		MD	University of Miami Miller School of	Miami, FL	Follow-up Examiner	NICHD Neonatal Research Network	
Silvia M.	Frade Eguaras		BA	University of Miami Miller School of	Miami, FL	Follow-up Examiner	NICHD Neonatal Research Network	
Andrea	Garcia		MS	University of Miami Miller School of	Miami, FL	Follow-up Examiner	NICHD Neonatal Research Network	
Michelle	Harwood Berkov		PhD	University of Miami Miller School of	Miami, FL	Follow-up Examiner	NICHD Neonatal Research Network	
Helina	Pierre		BA	University of Miami Miller School of	Miami, FL	Follow-up Examiner	NICHD Neonatal Research Network	
Arielle	Riguard		MD	University of Miami Miller School of	Miami, FL	Follow-up Examiner	NICHD Neonatal Research Network	
Alexandra	Stoerger		BA	University of Miami Miller School of	Miami, FL	Follow-up Examiner	NICHD Neonatal Research Network	
Lisa	Gaetano		RN MSN	Department of Pediatrics, Children's	Kansas City, MO, USA	Research Nurse	NICHD Neonatal Research Network	
Cheri	Gauldin		RN BSN CC	Department of Pediatrics, Children's	Kansas City, MO, USA	Research Coordinator	NICHD Neonatal Research Network	
Anne M.	Holmes		RN MSN M	Department of Pediatrics, Children's	Kansas City, MO, USA	Research Nurse	NICHD Neonatal Research Network	
Kathy	Johnson		RN CCRC	Department of Pediatrics, Children's	Kansas City, MO, USA	Research Nurse	NICHD Neonatal Research Network	
Howard W.	Kilbride		MD	Department of Pediatrics, Children's	Kansas City, MO, USA	Follow-up Principal Inv	NICHD Neonatal Research Network	
Eugenia K.	Pallotto		MD MSCE	Department of Pediatrics, University	Kansas City, MO, USA	Site Investigator	NICHD Neonatal Research Network	
Prabhu S.	Parimi		MD	Department of Pediatrics, University	Kansas City, MO, USA	Site Investigator	NICHD Neonatal Research Network	
Allison	Scott		RNC-NIC BS	Department of Pediatrics, Children's	Kansas City, MO, USA	Research Coordinator	NICHD Neonatal Research Network	

Supplemental Online Content: Nonauthor Collaborators
*First name, last name, and suffix (if applicable) are required and will appear in PubMed.

*First Name and Middle Initial(s)	*Last Name	*Suffix (eg, Jr, III)	Academic Degrees	Institution	Location (city, state/province, country)	Role or Contribution, eg, chair, principal investigator	Group (if more than 1 Group listed in the byline) and/or Subgroup (eg, Steering Committee)
William E.	Truog		MD	Department of Pediatrics, Children's	Kansas City, MO, USA	Principal Investigator	NICHD Neonatal Research Network
Gail E.	Besner		MD		Columbus, OH, USA	Surgeon	NICHD Neonatal Research Network
Erna	Clark		BA	Department of Pediatrics, Nationwid	Columbus, OH, USA	Research Assistant	NICHD Neonatal Research Network
Christine A.	Fortney		PhD RN	Department of Pediatrics, Nationwid	Columbus, OH, USA	Research Coordinator	NICHD Neonatal Research Network
Julie	Gutentag		RN BSN	Department of Pediatrics, Nationwid	Columbus, OH, USA	Research Nurse	NICHD Neonatal Research Network
Sudarshan R.	Jadcherla		MD FRCP (I	Department of Pediatrics, Nationwid	Columbus, OH, USA	Co-Principal Investigat	NICHD Neonatal Research Network
Patricia	Luzader		RN	Department of Pediatrics, Nationwid	Columbus, OH, USA	Research Coordinator	NICHD Neonatal Research Network
Nathalie L.	Maitre		MD PhD	Department of Pediatrics, Nationwid	Columbus, OH, USA		NICHD Neonatal Research Network
Leif D.	Nelin		MD	Department of Pediatrics, Nationwid	Columbus, OH, USA	Principal Investigator	NICHD Neonatal Research Network
Nehal A.	Parikh		DO MS	Department of Pediatrics, Nationwid	Columbus, OH, USA	Site Investigator	NICHD Neonatal Research Network
Courtney	Park		RN BSN	Department of Pediatrics, Nationwid	Columbus, OH, USA	Research Nurse	NICHD Neonatal Research Network
Pablo J.	Sánchez		MD	Department of Pediatrics, Nationwid	Columbus, OH, USA	Site Investigator	NICHD Neonatal Research Network
Julie C.	Shadd		BSN RD	Department of Pediatrics, Nationwid	Columbus, OH, USA	Research Assistant	NICHD Neonatal Research Network
Melanie	Stein		RRT BBA	Department of Pediatrics, Nationwid	Columbus, OH, USA	Research Assistant	NICHD Neonatal Research Network
Margaret	Sullivan		BS	Department of Pediatrics, Nationwid	Columbus, OH, USA	Research Assistant	NICHD Neonatal Research Network
Andrew A.	Bremer		MD PhD	Eunice Kennedy Shriver National Inst	Bethesda, MD, USA	Program Officer	NICHD Neonatal Research Network
Rosemary D.	Higgins		MD	Eunice Kennedy Shriver National Inst	Bethesda, MD, USA	Program Scientist	NICHD Neonatal Research Network
Stephanie	Wilson Archer		MA	Eunice Kennedy Shriver National Inst	Bethesda, MD, USA	Program Coordinator	NICHD Neonatal Research Network
Soraya	Abbasi		MD	Department of Pediatrics, University	Philadelphia, PA, USA	Site Investigator	NICHD Neonatal Research Network
Judy C.	Bernbaum		MD	Department of Pediatrics, University	Philadelphia, PA, USA	Follow-up Examiner	NICHD Neonatal Research Network
Christine	Catts		CRNP	Department of Pediatrics, University	Philadelphia, PA, USA	Research Assistant	NICHD Neonatal Research Network
Aasma S.	Chaudhary		BS RRT	Department of Pediatrics, University	Philadelphia, PA, USA	Research Coordinator	NICHD Neonatal Research Network
Noah	Cook		MD	Department of Pediatrics, University	Philadelphia, PA, USA		NICHD Neonatal Research Network
Dara M.	Cucinotta		RN	Department of Pediatrics, University	Philadelphia, PA, USA	Research Coordinator	NICHD Neonatal Research Network
Eric C.	Eichenwald		MD	Department of Pediatrics, University	Philadelphia, PA, USA	Principal Investigator	NICHD Neonatal Research Network
Marsha	Gerdes		PhD	Department of Pediatrics, University	Philadelphia, PA, USA	Follow-up Examiner	NICHD Neonatal Research Network
Sarvin	Ghavam		MD	Department of Pediatrics, University	Philadelphia, PA, USA	Site Investigator	NICHD Neonatal Research Network
Hallam	Hurt		MD	Department of Pediatrics, University	Philadelphia, PA, USA		NICHD Neonatal Research Network
Haresh	Kirpalani		BM MSc	Department of Pediatrics, University	Philadelphia, PA, USA	Co-Principal Investigat	NICHD Neonatal Research Network
Toni	Mancini		RN BSN CC	Department of Pediatrics, University	Philadelphia, PA, USA	Research Coordinator	NICHD Neonatal Research Network
Barbara	Schmidt		MD MSc	Department of Pediatrics, University	Philadelphia, PA, USA	Principal Investigator	NICHD Neonatal Research Network
Jonathan M.	Snyder		RN BSN	Department of Pediatrics, University	Philadelphia, PA, USA	Research Nurse	NICHD Neonatal Research Network
Sarith	Vangala		RN MSN	Department of Pediatrics, University	Philadelphia, PA, USA	Research Nurse	NICHD Neonatal Research Network

Supplemental Online Content: Nonauthor Collaborators
*First name, last name, and suffix (if applicable) are required and will appear in PubMed.

*First Name and Middle Initial(s)	*Last Name	*Suffix (eg, Jr, III)	Academic Degrees	Institution	Location (city, state/province, country)	Role or Contribution, eg, chair, principal investigator	Group (if more than 1 Group listed in the byline) and/or Subgroup (eg, Steering Committee)
Kristina	Ziolkowski		CMA(AAM/	Department of Pediatrics, University	Philadelphia, PA, USA		NICHD Neonatal Research Network
Julie	Babish Johnson		MSW	University of Rochester School of Me	Rochester, NY, USA	Follow-up Examiner	NICHD Neonatal Research Network
Kyle	Binion		BS	University of Rochester School of Me	Rochester, NY, USA	Research Assistant	NICHD Neonatal Research Network
Melissa	Bowman		RN NP	University of Rochester School of Me	Rochester, NY, USA		NICHD Neonatal Research Network
Erica	Burnell		RN	University of Rochester School of Me	Rochester, NY, USA	Research Nurse	NICHD Neonatal Research Network
Carl T.	D'Angio		MD	University of Rochester School of Me	Rochester, NY, USA	Principal Investigator	NICHD Neonatal Research Network
Caitlin	Fallone		MA	University of Rochester School of Me	Rochester, NY, USA		NICHD Neonatal Research Network
Osman	Farooq		MD	University of Rochester School of Me	Rochester, NY, USA		NICHD Neonatal Research Network
Ronnie	Guillet		MD PhD	University of Rochester School of Me	Rochester, NY, USA	Co-Principal Investigat	NICHD Neonatal Research Network
Cassandra A.	Horihan		MS	University of Rochester School of Me	Rochester, NY, USA	Research Coordinator	NICHD Neonatal Research Network
Julianne	Hunn		MSHCM	University of Rochester School of Me	Rochester, NY, USA		NICHD Neonatal Research Network
Diane	Hust		MS RN CS	University of Rochester School of Me	Rochester, NY, USA	Follow-up Coordinator	NICHD Neonatal Research Network
Rosemary L.	Jensen			University of Rochester School of Me	Rochester, NY, USA	Follow-up Coordinator	NICHD Neonatal Research Network
Rachel	Jones			University of Rochester School of Me	Rochester, NY, USA	Research Assistant	NICHD Neonatal Research Network
Jennifer	Kachelmeyer		BS	University of Rochester School of Me	Rochester, NY, USA	Research Assistant	NICHD Neonatal Research Network
Alison	Kent		BMBS FRA(	University of Rochester School of Me	Rochester, NY, USA	Site Investigator	NICHD Neonatal Research Network
Emily	Kushner		MA	University of Rochester School of Me	Rochester, NY, USA	Follow-up Examiner	NICHD Neonatal Research Network
Deanna	Maffett		RN	University of Rochester School of Me	Rochester, NY, USA	Research Nurse	NICHD Neonatal Research Network
Kimberly G.	McKee		MPH	University of Rochester School of Me	Rochester, NY, USA		NICHD Neonatal Research Network
Joan	Merzbach		LMSW	University of Rochester School of Me	Rochester, NY, USA	Follow-up Examiner	NICHD Neonatal Research Network
Gary J.	Myers		MD	University of Rochester School of Me	Rochester, NY, USA	Follow-up Principal Inv	NICHD Neonatal Research Network
Constance	Orme		BA	University of Rochester School of Me	Rochester, NY, USA	Research Assistant	NICHD Neonatal Research Network
Dale L.	Phelps		MD	University of Rochester School of Me	Rochester, NY, USA	Principal Investigator	NICHD Neonatal Research Network
Diane M.	Prinzing		AAS	University of Rochester School of Me	Rochester, NY, USA	Research Nurse	NICHD Neonatal Research Network
Linda J.	Reubens		RN CCRC	University of Rochester School of Me	Rochester, NY, USA	Research Coordinator	NICHD Neonatal Research Network
Daisy	Rochez		BS MHA	University of Rochester School of Me	Rochester, NY, USA	Research Assistant	NICHD Neonatal Research Network
Mary	Rowan		RN	University of Rochester School of Me	Rochester, NY, USA	Research Nurse	NICHD Neonatal Research Network
Premini	Sabaratnam		MPH	University of Rochester School of Me	Rochester, NY, USA	Research Assistant	NICHD Neonatal Research Network
Ann Marie	Scorsone		MS CCRC	University of Rochester School of Me	Rochester, NY, USA	Research Coordinator	NICHD Neonatal Research Network
Holly I.M.	Wadkins		MA	University of Rochester School of Me	Rochester, NY, USA	Research Coordinator	NICHD Neonatal Research Network
Kelley	Yost		PhD	University of Rochester School of Me	Rochester, NY, USA	Follow-up Examiner	NICHD Neonatal Research Network
Lauren	Zwetsch		RN MS PNF	University of Rochester School of Me	Rochester, NY, USA	Study Investigator	NICHD Neonatal Research Network
Carla M.	Bann		PhD	Social, Statistical and Environmental	Research Triangle Park, NC	Statistician	NICHD Neonatal Research Network

Supplemental Online Content: Nonauthor Collaborators
*First name, last name, and suffix (if applicable) are required and will appear in PubMed.

***First Name and Middle Initial(s)**	***Last Name**	***Suffix (eg, Jr, III)**	Academic Degrees	Institution	Location (city, state/province, country)	Role or Contribution, eg, chair, principal investigator	Group (if more than 1 Group listed in the byline) and/or Subgroup (eg, Steering Committee)	
Margaret M.	Crawford		BS CCRP	Social, Statistical and Environmental	Rockville, MD, USA	Research Coordinator	NICHD Neonatal Research Network	
Jenna	Gabrio		MPH CCRP	Social, Statistical and Environmental	Research Triangle Park, NC	Research Coordinator	NICHD Neonatal Research Network	
Marie G.	Gantz		PhD	Social, Statistical and Environmental	Research Triangle Park, NC	Statistician	NICHD Neonatal Research Network	
Betty K.	Hastings		AAS	Social, Statistical and Environmental	Research Triangle Park, NC	Research Coordinator	NICHD Neonatal Research Network	
David	Leblond		BS	Social, Statistical and Environmental	Research Triangle Park, NC	Database Programmer	NICHD Neonatal Research Network	
Jamie E.	Newman		PhD MPH	Social, Statistical and Environmental	Research Triangle Park, NC	Follow-up Coordinator	NICHD Neonatal Research Network	
Jeanette	O'Donnell Auma		BS	Social, Statistical and Environmental	Research Triangle Park, NC	Database Programmer	NICHD Neonatal Research Network	
Carolyn M.	Petrie Huitema		MS CCRP	Social, Statistical and Environmental	Rockville, MD, USA	Research Coordinator	NICHD Neonatal Research Network	
W. Kenneth	Poole		PhD	Social, Statistical and Environmental	Research Triangle Park, NC	Co-Principal Investigat	NICHD Neonatal Research Network	
Dennis	Wallace		PhD	Social, Statistical and Environmental	Research Triangle Park, NC	Co-Principal Investigat	NICHD Neonatal Research Network	
Kristin M.	Zaterka-Baxter		RN BSN CC	Social, Statistical and Environmental	Research Triangle Park, NC	Research Coordinator	NICHD Neonatal Research Network	
Michelle L.	Baack		MD	Department of Pediatrics, Sanford Sc	Sioux Falls, SD	Site Investigator	NICHD Neonatal Research Network	
Megan	Broadbent		RN BSN	Department of Pediatrics, Sanford Sc	Sioux Falls, SD	Research Nurse	NICHD Neonatal Research Network	
Chelsey	Elenkiwich		RN BSN	Department of Pediatrics, Sanford Sc	Sioux Falls, SD	Research Nurse	NICHD Neonatal Research Network	
Megan M.	Henning		RN	Department of Pediatrics, Sanford Sc	Sioux Falls, SD	Research Nurse	NICHD Neonatal Research Network	
Laurie A.	Hogden		MD	Department of Pediatrics, Sanford Sc	Sioux Falls, SD	Site Investigator	NICHD Neonatal Research Network	
Marian M.	Adams		MD	Department of Pediatrics, Division of	Palo Alto, CA, USA		NICHD Neonatal Research Network	
Dona	Bahmani		MD	Department of Pediatrics, Division of	Palo Alto, CA, USA		NICHD Neonatal Research Network	
M. Bethany	Ball		BS CCRC	Department of Pediatrics, Division of	Palo Alto, CA, USA	Research Coordinator	NICHD Neonatal Research Network	
Barbara	Bentley		PsychD MS	Department of Pediatrics, Division of	Palo Alto, CA, USA	Follow-up Examiner	NICHD Neonatal Research Network	
Elizabeth F.	Bruno		PhD	Department of Pediatrics, Division of	Palo Alto, CA, USA	Follow-up Examiner	NICHD Neonatal Research Network	
Valerie Y.	Chock		MD MS Epi	Department of Pediatrics, Division of	Palo Alto, CA, USA	Co-Principal Investigat	NICHD Neonatal Research Network	
Alexis S.	Davis		MD MS Epi	Department of Pediatrics, Division of	Palo Alto, CA, USA	Study Investigator	NICHD Neonatal Research Network	
Maria Elena	DeAnda		PhD	Department of Pediatrics, Division of	Palo Alto, CA, USA	Follow-up Examiner	NICHD Neonatal Research Network	
Anne M.	DeBattista		RN PNP-C F	Department of Pediatrics, Division of	Palo Alto, CA, USA	Follow-up Examiner	NICHD Neonatal Research Network	
Beth A.	Earhart		PsyD	Department of Pediatrics, Division of	Palo Alto, CA, USA		NICHD Neonatal Research Network	
Lynne C.	Huffman		MD	Department of Pediatrics, Division of	Palo Alto, CA, USA		NICHD Neonatal Research Network	
Magdy	Ismael		MD MPH	Department of Pediatrics, Division of	Palo Alto, CA, USA		NICHD Neonatal Research Network	
Jean G.	Kohn		MD MPH	Department of Pediatrics, Division of	Palo Alto, CA, USA	Follow-up Examiner	NICHD Neonatal Research Network	
Casey E.	Krueger		PhD	Department of Pediatrics, Division of	Palo Alto, CA, USA	Follow-up Examiner	NICHD Neonatal Research Network	
Julie C.	Lee-Ancajas		PhD	Department of Pediatrics, Division of	Palo Alto, CA, USA	Follow-up Examiner	NICHD Neonatal Research Network	
Janice	Lowe		MD	Department of Pediatrics, Division of	Palo Alto, CA, USA		NICHD Neonatal Research Network	
Ryan E.	Lucash		PhD	Department of Pediatrics, Division of	Palo Alto, CA, USA	Follow-up Examiner	NICHD Neonatal Research Network	

*First name, last name, and suffix (if applicable) are required and will appear in PubMed.

*First Name and Middle Initial(s)	*Last Name	*Suffix (eg, Jr, III)	Academic Degrees	Institution	Location (city, state/province, country)	Role or Contribution, eg, chair, principal investigator	Group (if more than 1 Group listed in the byline) and/or Subgroup (eg, Steering Committee)	
Andrew W.	Palmquist		RN BSN	Department of Pediatrics, Division of	Palo Alto, CA, USA	Research Nurse	NICHD Neonatal Research Network	
Jessica	Patel		PhD	Department of Pediatrics, Division of	Palo Alto, CA, USA		NICHD Neonatal Research Network	
Melinda S.	Proud		RCP	Department of Pediatrics, Division of	Palo Alto, CA, USA	Research Assistant	NICHD Neonatal Research Network	
Elizabeth N.	Reichert		MA CCRC	Department of Pediatrics, Division of	Palo Alto, CA, USA	Research Assistant	NICHD Neonatal Research Network	
Dharshi	Sivakumar		MD	Department of Pediatrics, Division of	Palo Alto, CA, USA	Follow-up Examiner	NICHD Neonatal Research Network	
Nicholas H.	St. John		PhD	Department of Pediatrics, Division of	Palo Alto, CA, USA	Follow-up Examiner	NICHD Neonatal Research Network	
David K.	Stevenson		MD	Department of Pediatrics, Division of	Palo Alto, CA, USA	Principal Investigator	NICHD Neonatal Research Network	
Heather L.	Taylor		PhD	Department of Pediatrics, Division of	Palo Alto, CA, USA		NICHD Neonatal Research Network	
Krisa P.	Van Meurs		MD	Department of Pediatrics, Division of	Palo Alto, CA, USA	Principal Investigator	NICHD Neonatal Research Network	
Natalie	Wager		PsyD	Department of Pediatrics, Division of	Palo Alto, CA, USA	Follow-up Examiner	NICHD Neonatal Research Network	
R. Jordan	Williams		BA	Department of Pediatrics, Division of	Palo Alto, CA, USA	Research Assistant	NICHD Neonatal Research Network	
Hali	Weiss		MD	Department of Pediatrics, Division of	Palo Alto, CA, USA		NICHD Neonatal Research Network	
Ana K.	Brussa		MS OTR/L	Department of Pediatrics, Division of	Boston, MA, USA	Follow-up Examiner	NICHD Neonatal Research Network	
Paige T.	Church		MD	Department of Pediatrics, Division of	Boston, MA, USA	Follow-up Principal Inv	NICHD Neonatal Research Network	
John M.	Fiascone		MD	Department of Pediatrics, Division of	Boston, MA, USA	Co-Principal Investigat	NICHD Neonatal Research Network	
Ivan D.	Frantz	III	MD	Department of Pediatrics, Division of	Boston, MA, USA	Principal Investigator	NICHD Neonatal Research Network	
Anne	Furey		MPH	Department of Pediatrics, Division of	Boston, MA, USA		NICHD Neonatal Research Network	
Anne	Kurfiss		MPH	Department of Pediatrics, Division of	Boston, MA, USA		NICHD Neonatal Research Network	
Brenda L.	MacKinnon		RN BSN	Department of Pediatrics, Division of	Boston, MA, USA	Research Coordinator	NICHD Neonatal Research Network	
Elisabeth C.	McGowan		MD	Department of Pediatrics, Division of	Boston, MA, USA	Follow-up Principal Inv	NICHD Neonatal Research Network	
Ellen	Nylen		RN BSN	Department of Pediatrics, Division of	Boston, MA, USA	Research Nurse	NICHD Neonatal Research Network	
Cecelia E.	Sibley		PT MHA	Department of Pediatrics, Division of	Boston, MA, USA	Follow-up Examiner	NICHD Neonatal Research Network	
Teresa	Chanlaw		MPH	Department of Pediatrics, University	Los Angeles, CA, USA	Research Coordinator	NICHD Neonatal Research Network	
Uday	Devaskar		MD	Department of Pediatrics, University	Los Angeles, CA, USA	Principal Investigator	NICHD Neonatal Research Network	
Meena	Garg		MD	Department of Pediatrics, University	Los Angeles, CA, USA	Co-Principal Investigat	NICHD Neonatal Research Network	
Rachel	Geller		RN BSN	Department of Pediatrics, University	Los Angeles, CA, USA	Research Coordinator	NICHD Neonatal Research Network	
Isabell B.	Purdy		PNP NNP P	Department of Pediatrics, University	Los Angeles, CA, USA	Follow-up Principal Inv	NICHD Neonatal Research Network	
Kathy	Arnell		RNC	Division of Neonatology, University c	San Diego, CA, USA	Research Coordinator	NICHD Neonatal Research Network	
Clarence	Demetrio		RN	Division of Neonatology, University c	San Diego, CA, USA	Research Coordinator	NICHD Neonatal Research Network	
Neil N.	Finer		MD	Division of Neonatology, University c	San Diego, CA, USA	Principal Investigator	NICHD Neonatal Research Network	
Martha G.	Fuller		PhD RN MS	Division of Neonatology, University c	San Diego, CA, USA	Follow-up Coordinator	NICHD Neonatal Research Network	
Christopher	Henderson		AS CRTT	Division of Neonatology, University c	San Diego, CA, USA	Research Coordinator	NICHD Neonatal Research Network	
David	Kaegi		MD	Division of Neonatology, University c	San Diego, CA, USA	Study Investigator	NICHD Neonatal Research Network	

Supplemental Online Content: Nonauthor Collaborators
*First name, last name, and suffix (if applicable) are required and will appear in PubMed.

*First Name and Middle Initial(s)	*Last Name	*Suffix (eg, Jr, III)	Academic Degrees	Institution	Location (city, state/province, country)	Role or Contribution, eg, chair, principal investigator	Group (if more than 1 Group listed in the byline) and/or Subgroup (eg, Steering Committee)	
Maynard R.	Rasmussen		MD	Division of Neonatology, University c	San Diego, CA, USA	Co-Principal Investigat	NICHD Neonatal Research Network	
Wade	Rich		BSHS RRT	Division of Neonatology, University c	San Diego, CA, USA	Research Coordinator	NICHD Neonatal Research Network	
Yvonne E.	Vaucher		MD MPH	Division of Neonatology, University c	San Diego, CA, USA	Follow-up Principal Inv	NICHD Neonatal Research Network	
Paul R.	Wozniak		MD	Division of Neonatology, University c	San Diego, CA, USA		NICHD Neonatal Research Network	
Janice	Bernhardt		MS RN	Division of Neonatal/Perinatal Medic	Chapel Hill, NC, USA	Research Coordinator	NICHD Neonatal Research Network	
Carl L.	Bose		MD	Division of Neonatal/Perinatal Medic	Chapel Hill, NC, USA	Site Investigator	NICHD Neonatal Research Network	
Gennie	Bose		RN	Division of Neonatal/Perinatal Medic	Chapel Hill, NC, USA	Research Coordinator	NICHD Neonatal Research Network	
Cynthia L.	Clark		RN	Division of Neonatal/Perinatal Medic	Chapel Hill, NC, USA	Research Coordinator	NICHD Neonatal Research Network	
Matthew Maxwell	Laughon		MD MPH	Division of Neonatal/Perinatal Medic	Chapel Hill, NC, USA	Site Investigator	NICHD Neonatal Research Network	
Diane D.	Warner		MD MPH	Division of Neonatal/Perinatal Medic	Chapel Hill, NC, USA	Follow-up Principal Inv	NICHD Neonatal Research Network	
Janice Kay	Wereszczak		CPNP-AC/P	Division of Neonatal/Perinatal Medic	Chapel Hill, NC, USA	Follow-up Examiner	NICHD Neonatal Research Network	
Conra	Backstrom Lacy		RN	University of New Mexico Health Scie	Albuquerque, NM, USA	Research Coordinator	NICHD Neonatal Research Network	
Andrea F.	Duncan		MD MScr	University of New Mexico Health Scie	Albuquerque, NM, USA	Follow-up Examiner	NICHD Neonatal Research Network	
Tara	Dupont		MD	University of New Mexico Health Scie	Albuquerque, NM, USA		NICHD Neonatal Research Network	
Janell	Fuller		MD	University of New Mexico Health Scie	Albuquerque, NM, USA	Follow-up Principal Inv	NICHD Neonatal Research Network	
Mary	Hanson		RN BSN	University of New Mexico Health Scie	Albuquerque, NM, USA	Research Nurse	NICHD Neonatal Research Network	
Carol H.	Hartenberger		MPH RN	University of New Mexico Health Scie	Albuquerque, NM, USA	Research Nurse	NICHD Neonatal Research Network	
Elizabeth	Kuan		RN BSN	University of New Mexico Health Scie	Albuquerque, NM, USA	Research Nurse	NICHD Neonatal Research Network	
Jean R.	Lowe		PhD	University of New Mexico Health Scie	Albuquerque, NM, USA	Follow-up Examiner	NICHD Neonatal Research Network	
Robin K.	Ohls		MD	University of New Mexico Health Scie	Albuquerque, NM, USA	Co-Principal Investigat	NICHD Neonatal Research Network	
Lu-Ann	Papile		MD	University of New Mexico Health Scie	Albuquerque, NM, USA	Co-Principal Investigat	NICHD Neonatal Research Network	
Sandra	Sundquist Beaun		MSN RNC-N	University of New Mexico Health Scie	Albuquerque, NM, USA	Research Coordinator	NICHD Neonatal Research Network	
Rebecca A.	Thomson		RN BSN	University of New Mexico Health Scie	Albuquerque, NM, USA	Follow-up Coordinator	NICHD Neonatal Research Network	
Kristi L.	Watterberg		MD	University of New Mexico Health Scie	Albuquerque, NM, USA	Principal Investigator	NICHD Neonatal Research Network	
John	Barks		MD	Department of Neonatal-Perinatal M	Ann Arbor, MI, USA	Site Investigator	NICHD Neonatal Research Network	
Martha D.	Carlson		MD PhD	Department of Neonatal-Perinatal M	Ann Arbor, MI, USA	Site Investigator	NICHD Neonatal Research Network	
Mary K.	Christensen		BA RRT	Department of Neonatal-Perinatal M	Ann Arbor, MI, USA	Research Coordinator	NICHD Neonatal Research Network	
Stephanie A.	Wiggins		MS	Department of Neonatal-Perinatal M	Ann Arbor, MI, USA	Research Coordinator	NICHD Neonatal Research Network	
Subrata	Sarkar		MD	Department of Neonatal-Perinatal M	Ann Arbor, MI, USA	Co-Principal Investigat	NICHD Neonatal Research Network	
Shawna	Baker		RN	Department of Pediatrics, Division of	Salt Lake City, UT, USA	Follow-up Coordinator	NICHD Neonatal Research Network	
Mariana	Baserga		MD MSCI	Department of Pediatrics, Division of	Salt Lake City, UT, USA	Co-Principal Investigat	NICHD Neonatal Research Network	
Karie	Bird		RN BSN	Department of Pediatrics, Division of	Salt Lake City, UT, USA		NICHD Neonatal Research Network	
Jill	Burnett		RNC BSN	Department of Pediatrics, Division of	Salt Lake City, UT, USA	Research Nurse	NICHD Neonatal Research Network	

Supplemental Online Content: Nonauthor Collaborators
*First name, last name, and suffix (if applicable) are required and will appear in PubMed.

***First Name and Middle Initial(s)**	***Last Name**	***Suffix (eg, Jr, III)**	Academic Degrees	Institution	Location (city, state/province, country)	Role or Contribution, eg, chair, principal investigator	Group (if more than 1 Group listed in the byline) and/or Subgroup (eg, Steering Committee)	
Susan	Christensen		RNC BSN	Department of Pediatrics, Division of	Salt Lake City, UT, USA	Research Nurse	NICHD Neonatal Research Network	
Laura	Cole Bledsoe		RN	Department of Pediatrics, Division of	Salt Lake City, UT, USA		NICHD Neonatal Research Network	
Sean D.	Cunningham		PhD	Department of Pediatrics, Division of	Salt Lake City, UT, USA	Follow-up Examiner	NICHD Neonatal Research Network	
Brandy	Davis		RN BSN	Department of Pediatrics, Division of	Salt Lake City, UT, USA	Research Nurse	NICHD Neonatal Research Network	
Jennifer O.	Elmont		RN BSN	Department of Pediatrics, Division of	Salt Lake City, UT, USA	Research Nurse	NICHD Neonatal Research Network	
Roger G.	Faix		MD	Department of Pediatrics, Division of	Salt Lake City, UT, USA	Principal Investigator	NICHD Neonatal Research Network	
Becky	Hall		APRN	Department of Pediatrics, Division of	Salt Lake City, UT, USA		NICHD Neonatal Research Network	
Erika R.	Jensen		APRN	Department of Pediatrics, Division of	Salt Lake City, UT, USA		NICHD Neonatal Research Network	
Jennifer J.	Jensen		RN BSN	Department of Pediatrics, Division of	Salt Lake City, UT, USA	Research Nurse	NICHD Neonatal Research Network	
Jamie	Jordan		RN BSN	Department of Pediatrics, Division of	Salt Lake City, UT, USA	Research Nurse	NICHD Neonatal Research Network	
Manndi C.	Loertscher		BS CCRP	Department of Pediatrics, Division of	Salt Lake City, UT, USA	Research Assistant	NICHD Neonatal Research Network	
Trisha	Marchant		RN BSN	Department of Pediatrics, Division of	Salt Lake City, UT, USA	Research Nurse	NICHD Neonatal Research Network	
Earl	Maxson		BSN	Department of Pediatrics, Division of	Salt Lake City, UT, USA	Research Nurse	NICHD Neonatal Research Network	
Kandace M.	McGrath		BS	Department of Pediatrics, Division of	Salt Lake City, UT, USA	Research Assistant	NICHD Neonatal Research Network	
Stephen D.	Minton		MD	Department of Pediatrics, Division of	Salt Lake City, UT, USA	Site Investigator	NICHD Neonatal Research Network	
Galina	Morshedzadeh		BSN APRN	Department of Pediatrics, Division of	Salt Lake City, UT, USA		NICHD Neonatal Research Network	
Karen A.	Osborne		RN BSN CC	Department of Pediatrics, Division of	Salt Lake City, UT, USA	Research Coordinator	NICHD Neonatal Research Network	
D. Melody	Parry		RN BSN	Department of Pediatrics, Division of	Salt Lake City, UT, USA	Research Nurse	NICHD Neonatal Research Network	
Carrie A.	Rau		RN BSN CC	Department of Pediatrics, Division of	Salt Lake City, UT, USA	Research Coordinator	NICHD Neonatal Research Network	
Brixen A.	Reich		MSN RNC (	Department of Pediatrics, Division of	Salt Lake City, UT, USA		NICHD Neonatal Research Network	
Susan T.	Schaefer		RRT RN BSI	Department of Pediatrics, Division of	Salt Lake City, UT, USA	Research Nurse	NICHD Neonatal Research Network	
Mark J.	Sheffield		MD	Department of Pediatrics, Division of	Salt Lake City, UT, USA	Site Investigator	NICHD Neonatal Research Network	
Cynthia	Spencer		RNC BSN	Department of Pediatrics, Division of	Salt Lake City, UT, USA	Research Nurse	NICHD Neonatal Research Network	
Michael	Steffen		PhD	Department of Pediatrics, Division of	Salt Lake City, UT, USA	Follow-up Examiner	NICHD Neonatal Research Network	
Kelly	Stout		PhD	Department of Pediatrics, Division of	Salt Lake City, UT, USA		NICHD Neonatal Research Network	
Ashley L.	Stuart		PhD	Department of Pediatrics, Division of	Salt Lake City, UT, USA		NICHD Neonatal Research Network	
Katherine	Tice		RN BSN	Department of Pediatrics, Division of	Salt Lake City, UT, USA	Research Nurse	NICHD Neonatal Research Network	
Kimberlee	Weaver-Lewis		RN MS	Department of Pediatrics, Division of	Salt Lake City, UT, USA	Research Nurse	NICHD Neonatal Research Network	
Sarah	Winter		MD	Department of Pediatrics, Division of	Salt Lake City, UT, USA	Follow-up Examiner	NICHD Neonatal Research Network	
Kathryn D.	Woodbury		RN BSN	Department of Pediatrics, Division of	Salt Lake City, UT, USA	Research Nurse	NICHD Neonatal Research Network	
Bradley A.	Yoder		MD	Department of Pediatrics, Division of	Salt Lake City, UT, USA	Principal Investigator	NICHD Neonatal Research Network	
Karen	Zanetti		RN	Department of Pediatrics, Division of	Salt Lake City, UT, USA		NICHD Neonatal Research Network	
Korinne	Chiu		MA	Wake Forest University School of Me	Winston-Salem, NC, USA	Follow-up Examiner	NICHD Neonatal Research Network	

Supplemental Online Content: Nonauthor Collaborators
*First name, last name, and suffix (if applicable) are required and will appear in PubMed.

*First Name and Middle Initial(s)	*Last Name	*Suffix (eg, Jr, III)	Academic Degrees	Institution	Location (city, state/province, country)	Role or Contribution, eg, chair, principal investigator	Group (if more than 1 Group listed in the byline) and/or Subgroup (eg, Steering Committee)
Robert G.	Dillard		MD	Wake Forest University School of Me	Winston-Salem, NC, USA	Follow-up Principal Inv	NICHD Neonatal Research Network
Deborah	Evans Allred		MA LPA	Wake Forest University School of Me	Winston-Salem, NC, USA	Follow-up Examiner	NICHD Neonatal Research Network
Donald J.	Goldstein		PhD	Wake Forest University School of Me	Winston-Salem, NC, USA	Follow-up Examiner	NICHD Neonatal Research Network
Raquel	Halfond		MA	Wake Forest University School of Me	Winston-Salem, NC, USA	Follow-up Examiner	NICHD Neonatal Research Network
Barbara G.	Jackson		RN BSN	Wake Forest University School of Me	Winston-Salem, NC, USA	Follow-up Coordinator	NICHD Neonatal Research Network
T. Michael	O'Shea		MD MPH	Wake Forest University School of Me	Winston-Salem, NC, USA	Principal Investigator	NICHD Neonatal Research Network
Nancy J.	Peters		RN CCRP	Wake Forest University School of Me	Winston-Salem, NC, USA	Research Coordinator	NICHD Neonatal Research Network
Carroll	Peterson		MA	Wake Forest University School of Me	Winston-Salem, NC, USA	Follow-up Examiner	NICHD Neonatal Research Network
Ellen L.	Waldrep		MS	Wake Forest University School of Me	Winston-Salem, NC, USA	Follow-up Examiner	NICHD Neonatal Research Network
Lisa K.	Washburn		MD	Wake Forest University School of Me	Winston-Salem, NC, USA	Follow-up Principal Inv	NICHD Neonatal Research Network
Cherrie D.	Welch		MD MPH	Wake Forest University School of Me	Winston-Salem, NC, USA	Follow-up Examiner	NICHD Neonatal Research Network
Melissa	Whalen Morris		MA	Wake Forest University School of Me	Winston-Salem, NC, USA	Follow-up Examiner	NICHD Neonatal Research Network
Gail	Wiley Hounshell		PhD	Wake Forest University School of Me	Winston-Salem, NC, USA	Follow-up Examiner	NICHD Neonatal Research Network
Stephen D.	Kicklighter		MD	Department of Pediatrics, Division of	Raleigh, NC, USA	Site Investigator	NICHD Neonatal Research Network
Ginger	Rhodes-Ryan		ARNP MSN	Department of Pediatrics, Division of	Raleigh, NC, USA	Research Coordinator	NICHD Neonatal Research Network
Donna	White		RN-BC BSN	Department of Pediatrics, Division of	Raleigh, NC, USA	Research Nurse	NICHD Neonatal Research Network
Katherine	Abramczyk			Department of Pediatrics, Wayne Sta	Detroit, MI, USA	Research Coordinator	NICHD Neonatal Research Network
Prashant	Agarwal		MD	Department of Pediatrics, Wayne Sta	Detroit, MI, USA	Site Investigator	NICHD Neonatal Research Network
Monika	Bajaj		MD	Department of Pediatrics, Wayne Sta	Detroit, MI, USA		NICHD Neonatal Research Network
Rebecca	Bara		RN BSN	Department of Pediatrics, Wayne Sta	Detroit, MI, USA	Research Coordinator	NICHD Neonatal Research Network
Elizabeth	Billian		RN MBA	Department of Pediatrics, Wayne Sta	Detroit, MI, USA	Research Coordinator	NICHD Neonatal Research Network
Sanjay	Chawla		MD	Department of Pediatrics, Wayne Sta	Detroit, MI, USA	Co-Principal Investigat	NICHD Neonatal Research Network
Kirsten	Childs		RN BSN	Department of Pediatrics, Wayne Sta	Detroit, MI, USA	Research Nurse	NICHD Neonatal Research Network
Lilia C.	De Jesus		MD	Department of Pediatrics, Wayne Sta	Detroit, MI, USA		NICHD Neonatal Research Network
Debra	Driscoll		RN BSN	Department of Pediatrics, Wayne Sta	Detroit, MI, USA		NICHD Neonatal Research Network
Melissa	February		MD	Department of Pediatrics, Wayne Sta	Detroit, MI, USA		NICHD Neonatal Research Network
Laura A.	Goldston		MA	Department of Pediatrics, Wayne Sta	Detroit, MI, USA	Follow-up Examiner	NICHD Neonatal Research Network
Mary E.	Johnson		RN BSN	Department of Pediatrics, Wayne Sta	Detroit, MI, USA	Research Nurse	NICHD Neonatal Research Network
Geraldine	Muran		RN BSN	Department of Pediatrics, Wayne Sta	Detroit, MI, USA	Research Coordinator	NICHD Neonatal Research Network
Girija	Natarajan		MD	Department of Pediatrics, Wayne Sta	Detroit, MI, USA	Principal Investigator	NICHD Neonatal Research Network
Bogdan	Panaitescu		MD PhD	Department of Pediatrics, Wayne Sta	Detroit, MI, USA	Site Investigator	NICHD Neonatal Research Network
Athina	Pappas		MD	Department of Pediatrics, Wayne Sta	Detroit, MI, USA	Follow-up Principal Inv	NICHD Neonatal Research Network
Jeannette E.	Prentice		MD	Department of Pediatrics, Wayne Sta	Detroit, MI, USA		NICHD Neonatal Research Network

Supplemental Online Content: Nonauthor Collaborators
*First name, last name, and suffix (if applicable) are required and will appear in PubMed.

***First Name and Middle Initial(s)**	***Last Name**	***Suffix (eg, Jr, III)**	Academic Degrees	Institution	Location (city, state/province, country)	Role or Contribution, eg, chair, principal investigator	Group (if more than 1 Group listed in the byline) and/or Subgroup (eg, Steering Committee)
Seetha	Shankaran		MD	Department of Pediatrics, Wayne Sta	Detroit, MI, USA	Principal Investigator	NICHD Neonatal Research Network
Beena G.	Sood		MD MS	Department of Pediatrics, Wayne Sta	Detroit, MI, USA	Co-Principal Investigat	NICHD Neonatal Research Network
Diane F.	White		RRT CCRP	Department of Pediatrics, Wayne Sta	Detroit, MI, USA	Research Nurse	NICHD Neonatal Research Network
Eunice	Woldt		RN MSN	Department of Pediatrics, Wayne Sta	Detroit, MI, USA		NICHD Neonatal Research Network
Christine G.	Butler		MD	Department of Pediatrics, Yale Unive	New Haven, CT, USA	Follow-up Coordinator	NICHD Neonatal Research Network
Patricia	Cervone		RN	Department of Pediatrics, Yale Unive	New Haven, CT, USA	Research Coordinator	NICHD Neonatal Research Network
Richard A.	Ehrenkranz		MD	Department of Pediatrics, Yale Unive	New Haven, CT, USA	Principal Investigator (	NICHD Neonatal Research Network
Patricia	Gettner		RN	Department of Pediatrics, Yale Unive	New Haven, CT, USA	Research Coordinator	NICHD Neonatal Research Network
Sheila	Greisman		RN	Department of Pediatrics, Yale Unive	New Haven, CT, USA	Follow-up Coordinator	NICHD Neonatal Research Network
Harris C.	Jacobs		MD	Department of Pediatrics, Yale Unive	New Haven, CT, USA	Site Investigator	NICHD Neonatal Research Network
Monica	Konstantino		RN BSN	Department of Pediatrics, Yale Unive	New Haven, CT, USA	Research Coordinator	NICHD Neonatal Research Network
JoAnn	Poulsen		RN	Department of Pediatrics, Yale Unive	New Haven, CT, USA	Research Nurse	NICHD Neonatal Research Network
Elaine	Romano		MSN	Department of Pediatrics, Yale Unive	New Haven, CT, USA	Follow-up Coordinator	NICHD Neonatal Research Network
Janet	Taft		RN BSN	Department of Pediatrics, Yale Unive	New Haven, CT, USA	Research Nurse	NICHD Neonatal Research Network
Joanne	Williams		RN BSN	Department of Pediatrics, Yale Unive	New Haven, CT, USA	Research Nurse	NICHD Neonatal Research Network
