# Supplementary material for: Incidence of and Neurodevelopmental Outcomes After Late-Onset Meningitis Among Children Born Extremely Preterm
Source: JAMA Netw Open. 2022 Dec 8;5(12):e2245826. doi: 10.1001/jamanetworkopen.2022.45826 (PMC9856224; doi:10.1001/jamanetworkopen.2022.45826)
Supplement: Supplement 3. — Data Sharing Statement [file jamanetwopen-e2245826-s003.pdf]

Data Sharing Statement

Brumbaugh. Incidence of and Neurodevelopmental Outcomes After Late-Onset Meningitis Among Children Born Extremely Preterm. *JAMA Netw Open*. Published December 08, 2022. doi:10.1001/jamanetworkopen.2022.45826

Data
Data available: Yes
Data types: Other (please specify)
Additional Information: Data reported in this paper may be requested through a data use agreement. Further details are available at https://neonatal.rti.org/index.cfm?fuseaction=DataRequest.Home.
How to access data: Data reported in this paper may be requested through a data use agreement. Further details are available at https://neonatal.rti.org/index.cfm?fuseaction=DataRequest.Home.
When available: With publication

Supporting Documents
Document types: None

Additional Information
Who can access the data: Data reported in this paper may be requested through a data use agreement. Further details are available at https://neonatal.rti.org/index.cfm?fuseaction=DataRequest.Home.
Types of analyses: Data reported in this paper may be requested through a data use agreement. Further details are available at https://neonatal.rti.org/index.cfm?fuseaction=DataRequest.Home.
Mechanisms of data availability: Data reported in this paper may be requested through a data use agreement. Further details are available at https://neonatal.rti.org/index.cfm?fuseaction=DataRequest.Home.
